# Neural Extrapolation of Motion for a Ball Rolling Down an Inclined Plane

**DOI:** 10.1371/journal.pone.0099837

**Published:** 2014-06-18

**Authors:** Barbara La Scaleia, Francesco Lacquaniti, Myrka Zago

**Affiliations:** 1 Laboratory of Neuromotor Physiology, IRCCS Santa Lucia Foundation, Rome, Italy; 2 Department of Systems Medicine, University of Rome Tor Vergata, Rome, Italy; 3 Centre of Space Bio-medicine, University of Rome Tor Vergata, Rome, Italy; University of California, Merced, United States of America

## Abstract

It is known that humans tend to misjudge the kinematics of a target rolling down an inclined plane. Because visuomotor responses are often more accurate and less prone to perceptual illusions than cognitive judgments, we asked the question of how rolling motion is extrapolated for manual interception or drawing tasks. In three experiments a ball rolled down an incline with kinematics that differed as a function of the starting position (4 different positions) and slope (30°, 45° or 60°). In Experiment 1, participants had to punch the ball as it fell off the incline. In Experiment 2, the ball rolled down the incline but was stopped at the end; participants were asked to imagine that the ball kept moving and to punch it. In Experiment 3, the ball rolled down the incline and was stopped at the end; participants were asked to draw with the hand in air the trajectory that would be described by the ball if it kept moving. We found that performance was most accurate when motion of the ball was visible until interception and haptic feedback of hand-ball contact was available (Experiment 1). However, even when participants punched an imaginary moving ball (Experiment 2) or drew in air the imaginary trajectory (Experiment 3), they were able to extrapolate to some extent global aspects of the target motion, including its path, speed and arrival time. We argue that the path and kinematics of a ball rolling down an incline can be extrapolated surprisingly well by the brain using both visual information and internal models of target motion.

## Introduction

Humans are generally accurate in the manual interception of an object dropped vertically from above, irrespective of whether the task involves catching [1]–[3], punching [4]–[8] or batting [9], [10]. The movements are time-locked to the expected arrival of the ball over a range of different heights of fall [1], [2], [5]. Given that the visuomotor delays for hand interceptions are considerable (100–300 ms, see [3], [11]–[13] and the visual estimates of image acceleration are generally poor [11], [14]–[17], the reported motor accuracy depends on mechanisms extrapolating visual motion forward in time by means of an internal model of gravity effects [8], [15], [18]–[27].

Vertical free-falls represent the simplest case of gravitational motion, because the gravitational acceleration is nearly constant (*g*≈9.8 m s^−2^) on the Earth’s surface and the effects of non-gravitational forces (such as air drag) can be neglected for many interceptive actions [19], [26]. It remains unclear how humans deal with more complex gravitational kinematics. One experimental strategy to address the problem of the predictive power of a model of gravity effects is to follow Galileo and change systematically the net acceleration due to gravity by means of an inclined plane [28]. For instance, an object slides down a frictionless plane with acceleration equal to *g*sin*α*, where *α* is the inclination angle relative to the horizontal. If instead a homogeneous spherical object (such as a ball) rolls down without slipping, its acceleration is (5/7)*g*(sin*α*–*µ_v_*cos*α*/*R*), where *µ_v_* is the coefficient of rolling resistance and *R* the object radius (see **Appendix**). Are we able to cope with accelerations that vary to such a large extent as a function of the inclination angle and surface/object properties? Does the internal model of gravity generalize to conditions with fractional gravity effects?.

The answer to both questions would seem to be negative based on studies which probe the explicit awareness of the effects of gravity on sliding and rolling [29]. In such cases, perceptual judgments tend to be systematically flawed. Bozzi [30], [31] studied the perception of sliding motion along a plane inclined at different angles. He projected artificially generated motions of a square target on a screen, and asked observers to choose the motion function which looked like a natural, frictionless sliding down the incline among several alternatives. He found that sliding is perceived as natural not when it is uniformly accelerated, as expected from physics, but when the object accelerates at the start and then moves at constant speed for the rest. Hecht [32] used computer-generated displays of wheels rolling down an inclined plane to address the issue of whether observers are able to appreciate the kinematic coupling of rotation and translation, and the dynamic effects of gravity. He found that observers are unable to differentiate between different acceleration functions, and their judgments are based on the translation component of the rolling motion, while rotation is neglected. Vicario and Bressan [33] described a related illusion: wheels appear to revolve much faster than is compatible with their linear translation. Also the memory of the final position for an object rolling down an inclined plane without friction is distorted and the extent of the distortion depends strongly on the slope [28]. Finally, people (adolescents) asked to imagine a trolley freely rolling down along an incline, once provided with information about total travel time, estimate the traveled distance under the assumption of constant speed motion, instead of accelerated motion [34].

According to Proffitt and Gilden [35], people make judgments about natural object motions on the basis of only one parameter of information that is salient in the event, and encounter difficulties when evaluating the dynamics of any mechanical system that has more than one dynamically relevant parameter. This accounts for the very poor understanding of wheel dynamics [36]. Notice, though, that the visual system can use contextual cues, along with intrinsic surface cues, to compute percepts of rolling objects [37].

On the other hand, it is well known that automatic visuomotor responses generally are much more accurate and less prone to perceptual illusions than cognitive judgments about the same targets, consistent with the idea that visual information for action and for explicit awareness are processed in a distinct manner (e.g., [38]–[40]). Thus, perceived slant of an incline is grossly overestimated, whereas the corresponding action measures are accurate [41], [42]. Moreover, rolling objects with rotational and translational motion congruent with an object rolling on the ground elicit faster tracking eye movements during pursuit initiation than incongruent stimuli [43].

To our knowledge, only one study dealt with the manual interception of an object shifting along slanted trajectories, and it relied on simulated motion in a virtual reality setup [44]. In this study, participants were asked to abduct their index finger at the time of arrival of a ball rolling within a tube which could have various shapes. On average, participants responded too early and their errors varied with the slope of the tube, indicating that interception was not accurate. Moreover, the errors were similar when the tube was invisible depriving subjects of any cue that could be used to predict the target trajectory in advance, and when the visual scene was projected upside down violating ecological gravity constraints. This indicates that the task could be accomplished, to a certain degree, by visual online feedback only. However, the errors in catch trials, in which the speed of the ball was unexpectedly maintained constant, differed depending on whether the tube was visible or not. This pattern of errors suggested that participants learned to predict some effects of acceleration normally induced by the shape of the tube within the practice period. It should be noticed that, because the virtual scene did not include any scaling or texture cues, the dynamic parameters (gravity, mass, friction) were undetermined, and this might explain the presence of both bias and variability in the performance, as well as the heavy reliance on visual online feedback [44].

We examined the issue by investigating the interception of a real object rolling down an inclined plane. Both visual perception and motor interaction involving real objects can differ substantially from those involving virtual objects [45]–[47]. In particular, interception of a target falling vertically [6] or swinging as a pendulum [45] under gravity is more accurate with a real ball than with a virtual target. Moreover, the role of internalized laws of dynamics may become more relevant in natural environments complying with the ecological constraints of the real world [48].

Here we performed three experiments in which a ball rolled down an incline with kinematics that differed as a function of the starting position and slope. In Experiment 1, participants were asked to punch the ball after its exit from the incline. In Experiment 2, the ball rolled down the incline but was stopped at the end; participants were asked to punch the imaginary ball as if it continued its motion. In Experiment 3, the ball rolled down the incline and was stopped at the end, as in Experiment 2, but here participants were asked to draw with the hand in air the trajectory that would be described by the ball if it kept moving. By varying the motor task and/or the feedback about the terminal part of ball trajectory across experiments, we aimed at elucidating the nature of the predictive processes involved in the extrapolation of the ball trajectories.

## Experiment 1

In this experiment, participants intercepted a ball falling from an inclined plane. We changed the slope *α* of the incline in different blocks of trials, and randomized the starting position of the ball on the incline (resulting in different durations of ball motion) across trials. Accordingly, ball acceleration on the incline and speed at the time of interception varied by about two times over the range of conditions. To impose stringent margins of spatial and temporal accuracy, we constrained the valid interception region to a small volume along the path of fall of the ball. In this manner, we were also able to match the duration of ball motion prior to interception across all tilt angles.

Different hypotheses about the law of ball motion which is assumed by the subjects make different predictions about the timing of the interceptive responses. If subjects assumed constant speed motion, they would arrive too late at the interception point and miss the ball. If instead they assumed constant acceleration equal to Earth gravity for all tilt angles, they would arrive too early. They would also arrive too early if they took into account the translational but not the rotational component of ball motion. Only if all components of rolling motion were taken into account, would interceptions be timed correctly independent of tilt angle.

### Methods

#### Participants

All participants in this and the following experiments gave written informed consent to procedures approved by the Institutional Review Board of Santa Lucia Foundation, in conformity with the Declaration of Helsinki on the use of human subjects in research. All participants were right-handed (as assessed by a short questionnaire based on the Edinburgh scale), had normal or corrected-to-normal vision, no past history of psychiatric or neurological diseases, and were naïve to the specific purpose of the experiments. Fifteen subjects (11 females and 4 males, 28±7 years old, mean ± SD) participated in Experiment 1.

#### Experimental set-up

**Figure 1:**
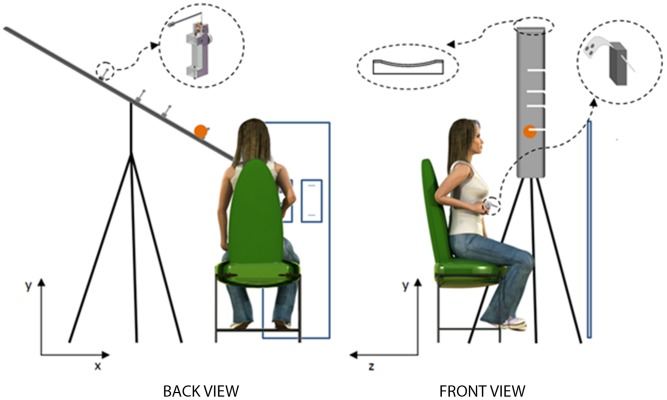
Schematic of the experimental setup. For simplicity, only one tripod stand is depicted (instead of the 3 tripods actually used).

Subjects sat on a chair placed in front of a vertical projection screen (in the following denoted as “screen”; Fig. 1), and in front and to the right of the inclined surface (in the following denoted as “incline”) from which a ball rolled down (Fig. S1). The screen was 0.45 m wide and 1.4 m high. The incline was 2.1 m long and 0.2 m wide. Its surface was made of inox steel, and served as the track for the rolling ball. The surface had been especially machined so as to be very smooth (see **Appendix** for estimates of dynamic friction). It was glued as a groove to a 6-cm thick laminated poplar, and the whole structure was supported by three tripod stands. The longitudinal axis of the incline and thus the direction of ball descent were roughly parallel to the frontal plane of the subject. By changing the height of the tripods and the orientation of their joints, the incline could be tilted relative to the horizontal by one of 3 angles (30°, 45°, or 60°) in different blocks of trials. Irrespective of the tilt angle, the lower end of the incline and exit point of the ball were at 0.81 m height above the floor. A soft, homogenous rubber ball (diameter, 9 cm; weight, 30.2 g) rolled down the incline with different accelerations depending on the starting position and tilt angle, without slipping or bouncing. After exiting from the lower end of the incline, the ball fell under gravity and air drag along a quasi-parabolic trajectory. Notice that the homogeneous surface of the balls provided minimal optical cues about the rotational component of the motion.

The incline was equipped with 12 electromechanical lever arms spaced along the track, which allowed the release of the ball from different starting positions (see **Protocol**). These devices were located on the side of the incline opposite to that facing the subject; consequently, they did not prevent the view of the descending ball nor did they interfere with ball motion. Each lever consisted of a thin steel rod whose position (up or down) was rapidly set by a solenoid (G.W. Linsk, Clifton Springs, NY) under computer control. The time accuracy of ball release was better than 1 ms. An on-axis laser transmitter/receiver (transceiver) was placed at 5 cm distance from the lower end of the incline, orthogonal to the direction of ball descent, to monitor the descent time of the ball in each trial.

The back of the chair, supporting the head and torso of the subject, was adjusted at about 25° relative to the vertical, allowing a comfortable position and full view of the incline. Subject’s eyes were at a horizontal distance of about 50 cm from the incline longitudinal axis, 8 cm to the right and 31 cm above the lower end of the incline. Their horizontal distance from the screen was about 1.2 m. By protracting the arm forwards, the subject could easily reach the interception region just below and to the right of the lower end of the incline.

Subjects held an instrumented plastic box (size: 2×6.5×3.2 cm [w×h×d]; weight: 65 g) in their right hand. The box had a protruding steel pin (4.2 cm length, 0.4 cm diameter) on the front side, and a steel winged frame fixed over the horizontal side. The box was grasped by the subject so that the pin (in the following denoted as “hitter”) protruded between the index and middle finger, and the winged frame was roughly parallel to the back of the hand (Fig. 1, inset). Inside the box there was a tri-axial piezoelectric accelerometer which measured box (and hand) acceleration (Isotron 63-B100, Endevco, San Juan Capistrano, CA; ±50 g dynamic range, amplitude nonlinearity <1%). The accelerometer and the laser receiver were sampled by the acquisition system at 1 kHz. The position of the box in 3D was recorded at 200 Hz by means of the Optotrak 3020 system (Northern Digital, Waterloo, Ontario; ±3 SD-accuracy better than 0.2 mm for x, y, z coordinates). To this end, three infrared emitting markers were attached to the winged frame of the box. The 3D position of the tip of the hitter was derived from the known geometry of the box plus hitter, and the measured 3D position and orientation of the box. Three additional markers were placed on the incline to determine its position and orientation, one at 80 cm and the other two at 1 cm distance from the lower end of the incline.

Synchronous acquisition of all motion data (accelerometer, Optotrak, and laser receiver) was accomplished by the real-time system PXI-1010 (National Instruments, Austin, Texas) programmed with custom-made software. In each trial, data acquisition was started by the experimenter about 250 ms before ball release and lasted 2 s.

#### Task

Subjects were asked to punch the ball falling from the incline with the hitter. The ball had to be hit at the time its center reached the nominal interception point (nIP) along the quasi-parabolic path. nIP was located 5.5 cm below, and 13.5, 11 or 8 cm to the right of the lower end of the incline, for tilt angles of 30°, 45° or 60°, respectively. We provided continuous feedback of task variables by projecting on the screen in front of the subject 1) the time-varying position of the hitter, 2) the nominal starting position of the hitter (nSP) fixed for each trial, 3) the nominal interception point (nIP) fixed for each trial, and 4) the position of the lower end of the incline. Two 2D-projections with a front-view and a top-view of these points were shown side by side on the screen. The position of the box with the hitter was updated in real time (within 0.5 ms) by means of the acquired Optotrak data. nSP and nIP were shown inscribed within a 2 cm side square (green for nSP and red for nIP).

Subjects kept a relaxed posture between trials. Shortly before trial start, they were asked to look at the starting position of the ball on the incline and at the corresponding nIP. They were prompted to recoil the arm in the starting posture so as to place the tip of the hitter within the 2-cm-square around nSP. With the adducted shoulder, the upper arm was roughly vertical, the forearm horizontal, the wrist mid-pronated, the hand and fingers clenched around the box. In this way, the nSP was located 12.5 cm below and 13.5, 11 or 8 cm to the right of the lower border of the incline, for tilt angles of 30°, 45° or 60°, respectively. The initial horizontal distance between the nominal starting position of the hitter tip and the frontal plane passing through the longitudinal axis of the incline was 20 cm.

After a pseudo-random delay of 100, 200, 300 or 400 ms, the ball was released and rolled down the incline with the kinematics described in the **Appendix**. Apart from the on-line feedback on the screen and the direct sensory information about ball trajectory, no further information was provided to the participants about their performance in hitting the ball.

#### Protocol

In separate blocks of trials, the incline was tilted by 30°, 45°, or 60° (order counterbalanced across participants). Ball acceleration was roughly constant on the plane for a given inclination, being 3.21 m·s^−2^, 4.71 m·s^−2^ and 5.90 m·s^−2^ for tilt angles of to 30°, 45° and 60°, respectively, while it was 9.81 m·s^−2^ during the free-fall phase of all tilts (see **Appendix**). At each tilt angle, there were 4 possible starting points of the ball along the incline (different among tilt angles), resulting in 4 nominal durations of ball motion (nBMD, see Table S1 and Fig. S1). nBMD was the total motion duration from release to nIP and was matched across tilt angles. nBMD was randomized across trials, avoiding identical values in consecutive trials. In a block of trials, each nBMD was presented 15 times for a total of 60 trials at each tilt angle. Thus, one experiment included 180 trials (60 trials×3 tilt angles). Participants were allowed to pause any time they wished during the experiment. Total duration of an experiment was about 2 hours.

#### Data analysis

Out of a total of 2700 trials (180 trials×15 subjects), 60 were excluded (2.2%) from the analysis due to the presence of artifacts or lack of subject’s attention (as marked in the experiment’s notebook). When the latter event occurred, the subject was allowed to pause briefly so as to recover full attention to the task. Except for the detection of contact events (see below), raw kinematic data were numerically low-pass filtered (bi-directional, 25-Hz cutoff, 2^nd^ order Butterworth filter) to eliminate high-frequency oscillations due to contact. The instantaneous velocity of the hitter was estimated by numerically differentiating (finite difference approximations) the recorded x, y, z coordinates.

*Endpoint analysis*. We evaluated the position of the hitter when it first reached the minimum distance from the nominal interception point nIP. This position is denoted interception point (IP) in the following.

*Timing error* . For each trial, we computed the timing error (TE) as the difference between the time of arrival of the hitter in IP and nBMD. A positive value of TE corresponds to a response later than that theoretically expected (and a negative value to an earlier response) if the hitter arrived at IP at the same time as the center of the ball. Mean TE and 95% confidence interval were computed over all trials of each condition (tilt angle and nBMD). The theoretical margin of error for the timing of successful hitting movements through nIP was on the order of±25 ms, depending on the velocity of the ball. In fact, the actual margin was larger because the ball could be hit at any point on its surface and different points along the descent.

To characterize the rate at which subjects improved their timing during an experiment, we fitted an exponential function to the series of TE values across successive trials (non-linear least-squares fitting):
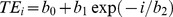
(1)where TE*_i_* is the TE value for repetition i, while *b_0_*, *b_1_*, and *b_2_* are the offset, gain, and learning-constant.

As an additional estimate of the timing errors, we used a spatiotemporal criterion that takes into account the physical dimensions of the ball. Accordingly, we considered the time at which the hitter first arrived at the minimum distance from the surface of the ball with its center in nIP, and compared it with the actual arrival time of the ball in the same position.

Hitter position. For each trial, we computed the spatial error as the Euclidean distance between IP values and nIP. Mean spatial error and 95% confidence interval were computed over all trials of each condition (tilt angle and nBMD). In addition, we computed the 2D spatial distribution of IP in the plane of the ball’s flight for all trials of each condition.

For a given tilt angle α and nominal ball motion duration nBMD, let 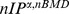 denote the nominal interception point and 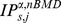 the position, for trial *j* of subject *s*, of the hitter-tip when it first reached the minimum distance relative to the nominal interception point nIP (i.e. at interception time). At interception point IP, the systematic spatial bias 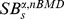 of subject *s* is characterized in terms of the 2D vector difference between the hitter-tip position at interception (averaged across all repetitions of nBMD) and nominal interception point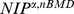:
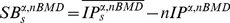
(2)


The values obtained in all individual subjects were averaged to yield the overall systematic spatial bias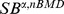.

The deviation δ from the mean for a given trial *j* of subject *s* is defined as
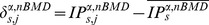
(3)


The 2×2 matrix of variance and covariance (hereafter referred to as the covariance matrix) of subject *s* is expressed by the equation:
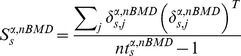
(4)where 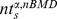 represents the number of trials of subject 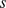 for angle 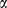 and nominal ball motion duration 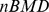 and *T* denotes the transpose.

By pooling the interception points across all subjects, the estimate of the combined covariance matrix for angle 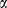 and 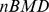 is:
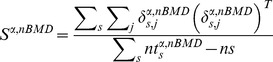
(5)where 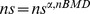 is the number of subjects (*ns* = 15).

The spatial precision (variability of performance) of all subjects for tilt angle 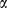 and 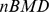 was computed by means of the 95% tolerance ellipse for each condition. This ellipse, based on the data from all trials of all subjects for a given condition, is obtained by scaling the combined covariance matrix:
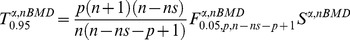
(6)where 
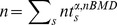
 is the total number of trials of all subjects for a given condition, and *p* ( = 2) is the dimensionality of the Cartesian vector space [49].

Eigenvalues and eigenvectors of the 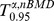 matrix were used to characterize the shape, size and orientation of the ellipse [50], [51]. The two axes of the ellipse have the same orientation as the two eigenvectors of 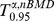 matrix, and the lengths of the two semi-axes correspond to the square roots of the two corresponding eigenvalues. We tested whether the eigenvalues were statistically different between each other by means of a 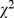 test at 95% confidence level 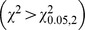, where 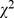 has the form ([51], p. 336):
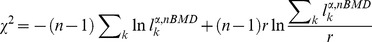
(7)where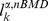 are the 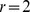 eigenvalues being compared for a given covariance matrix 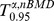 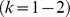. 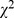 has 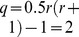 degrees of freedom.

Contact. To analyze the contact between the ball and the hitter (or hand/box), the raw data from the accelerometer were filtered with a bidirectional 25-Hz high-pass Butterworth filter. Visual inspection of the filtered accelerometer traces in single trials allowed to recognize contact by the presence of a brief burst of high-frequency oscillations [7]. Contact time denotes the time of occurrence of these oscillations. Detailed visual inspection of the combined data set of hitter position (measured with Optotrak) and acceleration (measured with the accelerometer) showed that, when the hitter passed close to the nIP, contact oscillations almost invariably started within the time interval between the positive peak and the negative peak of acceleration, consistent with previous work on ball punching [6], [7]. The contact oscillations started outside this interval in only 3.38% of all trials. Accordingly, we assigned a score of 1 to trials in which the contact oscillations started within the time interval defined above, and a score of 0 if there were no detectable oscillations or they started outside that interval. The contact rate was then computed as the percentage of all trials scoring 1 over the total number of trials of all subjects.

*Hitting movement.* We estimated the onset time of hitter movement (MOT, time when the hitter-tip speed first reached 0.05 m·s^−1^) and movement duration MD (interval between MOT and hitter arrival time in IP).

Tangential velocity (denoted as “speed” in the following) of the hitter was computed as 
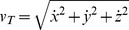
. Initial hitter speed (ISpeed) was defined as the average speed of the hitter in a window of 100 ms starting from MOT. We measured also the peak of hitter speed (PSpeed) during the hitting movement and the hitter speed in IP (Speed_IP). The curvature of hitter trajectory (TCurv) was defined as the mean of the perpendicular distances of all sampled points along the hitter trajectory (from movement onset to IP) relative to a straight line connecting the position of the hitter at movement onset and IP [52].

#### Statistics

Differences between conditions were assessed using Repeated Measures ANOVA (RM-ANOVA with tilt angle, nBMD and repetition as within-subjects factors) with post-hoc Bonferroni corrections for multiple comparisons (P<0.05).

### Results and Discussion

#### Interception performance

**Figure 2:**
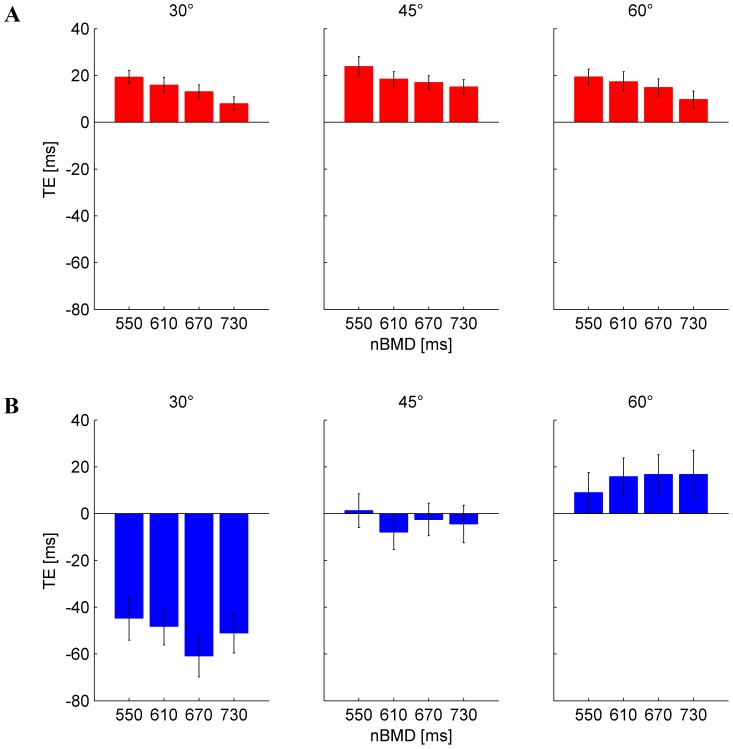
Timing error TE for each condition (tilt angle and duration of ball motion nBMD). Mean±95% confidence interval, over all subjects and repetitions (n = 225, except for missing trials, see text). A) Results from Experiment 1. B) Results from Experiment 2.

Interception timing was often close to ideal (i.e., timing error TE = 0). The mean TE (computed over all repetitions of all conditions) was 16 ms (SD = 26 ms, n = 2640, Fig. 2A), within the theoretical margin of error (see **Methods**). Three-way RM-ANOVA (3 tilt angles×4 nBMDs×15 repetitions) showed that TE depended significantly on the nominal duration of ball motion nBMD (F_3,42_ = 19.91, P<0.001) and repetitions (F_14,196_ = 4.321, P<0.001), while it was independent of tilt angle and any interaction between factors (P>0.092). In particular, TE tended to decrease with increasing nBMD and repetition. Post-hoc tests showed that TE for the longest duration (nBMD = 730 ms) was significantly smaller than that for the other nBMDs, while TE for the first repetition was significantly higher than that in all subsequent repetitions (all P<0.05) except repetition 2, 4, and 6.

**Figure 3:**
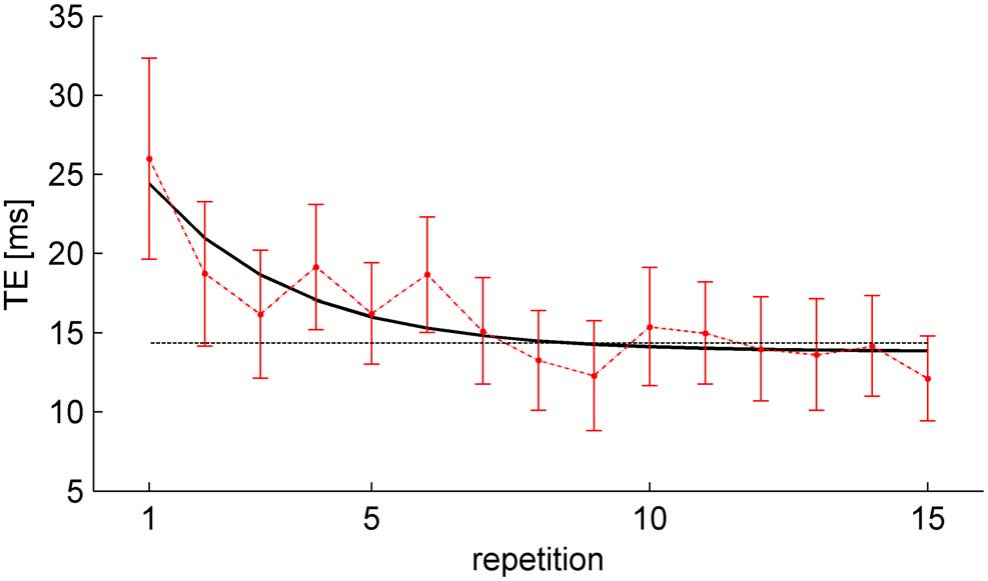
Experiment 1. Effect of practice on timing errors. Mean TE (±95% confidence interval, over all subjects, tilt angles and nBMD, n = 180) is plotted for each repetition (red), together with the exponential best-fit (black), and the 95% decrement (dotted gray).

Neither the mean TE computed over the first repetition of all conditions nor the mean TE computed over the first 3 repetitions of all conditions depended significantly on tilt angle, nBMD or interactions (all P>0.1). The effect of practice on timing was quantified by best-fitting (r^2^ = 0.731) eq. 1 to the TE of all subjects and conditions as a function of repetition (Fig. 3). We found a short learning constant (*b_2_* = 2.532, 95% confidence limits: [0.5104, 4.563]), indicating that learning occurred over the first few repetitions: TE was reduced by 95% of the overall change relative to the steady state value after only 7 repetitions. The other fitting parameters were: offset *b_0_* = 0.01383 ms (95% confidence limits: [0.01205, 0.0156] ms) and gain *b_1_* = 0.0106 ms (95% confidence limits: [0.0069, 0.0143] ms).

Qualitatively similar results were obtained by using an alternative estimate of the timing errors that takes into account the physical dimensions of the ball (see **Methods**). The mean difference between the time at which the hitter was closest to the position of the ball surface and the actual arrival time of the ball, computed over the last 8 repetitions (steady-state) of each condition, was 1±21 ms, 6±23 ms and 4±20 ms for tilt angles equal to 30°, 45° and 60° respectively. These timing errors are even smaller than the TE values reported above, and confirm the high spatiotemporal accuracy of the interceptive actions at steady-state.

**Figure 4:**
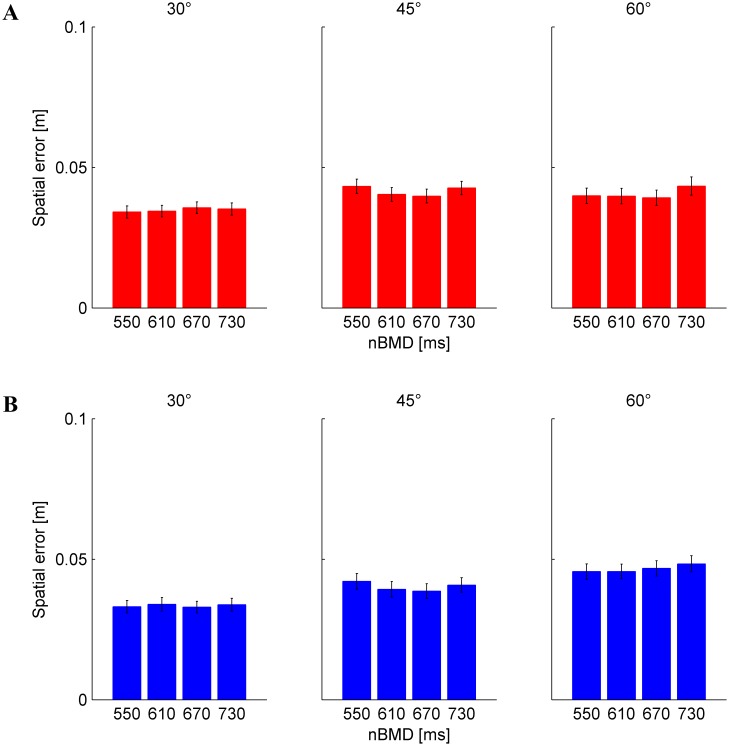
Spatial error (mean±95% confidence interval, over all subjects and repetitions, n = 225) for each condition (tilt angle and nBMD). A) Results from Experiment 1. B) Results from Experiment 2.

In agreement with this conclusion, we also found that the mean spatial error (distance between IPs and nIP) was 3.90 cm (SD = 1.93 cm, n = 2640), smaller than the ball radius (4.5 cm). Spatial error (Fig. 4A) depended slightly but significantly on tilt angle (3-way RM-ANOVA, 3 angles×4 nBMDs×15 repetitions (F_2,28_ = 4.786, P = 0.016), while it was independent of nBMD, repetitions, and any interactions (P>0.093). Post-hoc tests revealed that the spatial error was significantly smaller for 30° than 45° angle (P = 0.02).

**Figure 5:**
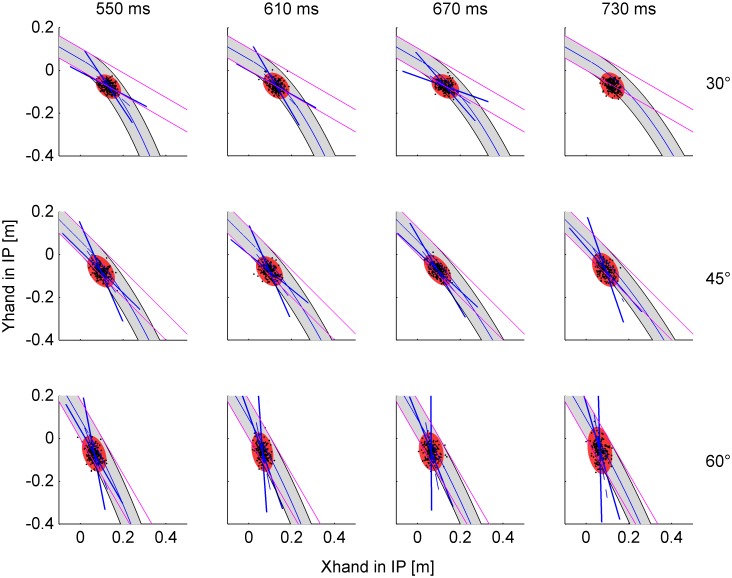
Experiment 1. Spatial distribution of interception points. IPs in single trials (black dots) and 95%-tolerance ellipses (red) for each condition. The first eigenvector (along the major axis of the ellipse) with the 95%-confidence cone (blue straight lines) is drawn when significant (see text). The gray area within black curved lines represents the envelope of the ball trajectory, and the blue curved line is the trajectory of the ball center. The pink straight lines represent the linear trajectory that would be followed by the ball if it continued the motion on the extended inclined plane.

The scatter of interception points across trials was not simply due to random variability, but was accounted for, at least in part, by the attempt of the subjects to intersect the ball trajectory at a point placed along the path of the ball. This is demonstrated by the statistical analysis of the spatial distribution of IPs in single trials. To this end, we computed the tolerance ellipses including 95% of IPs (T*_0.95_*) for each experimental condition (n = 12, 4 nBMD×3 angles). These ellipses (red ovoids in Fig. 5) are projected in the plane of motion of the ball’s center and are centered on the mean IP for any given condition. The magnitude of the variable error is provided by the square root of the eigenvalues of T*_0.95_* (see **Methods**). We found that 11 out of 12 ellipses had statistically different eigenvalues (Table S2), implying that the distribution of positions was not isotropic but tended to align along specific directions. Critically, the 95% confidence limits of the first eigenvector (aligned with the major axis of the ellipse, Table S3) included the tangent in the mean IP to the trajectory of the ball in several conditions: all nBMDs at 30° tilt angle (although the two eigenvalues were not significantly different for nBMD = 730 ms, 30° tilt), the 3 shortest nBMDs at 45°, and the shortest nBMD at 60°. In all such conditions, the individual responses tended to be distributed along the ball trajectory, consistent with the hypothesis that subjects were able to predict the trajectory. In this regards, notice that the tolerance ellipses overlap appreciably more with the parabolic trajectories than with the linear trajectories which would be followed by the ball if it continued rolling on an extended inclined plane for 30° and 45° tilt (Fig. 5). For instance, the major axis of the ellipse for 30° tilt, nBMD = 610 ms and for 45° tilt, nBMD = 730 ms deviated by about 15° from the linear path. Instead, the ellipses overlap with both parabolic and linear trajectories for 60° tilt, because these two sets of trajectories diverge further down at 60°-tilt.

**Figure 6:**
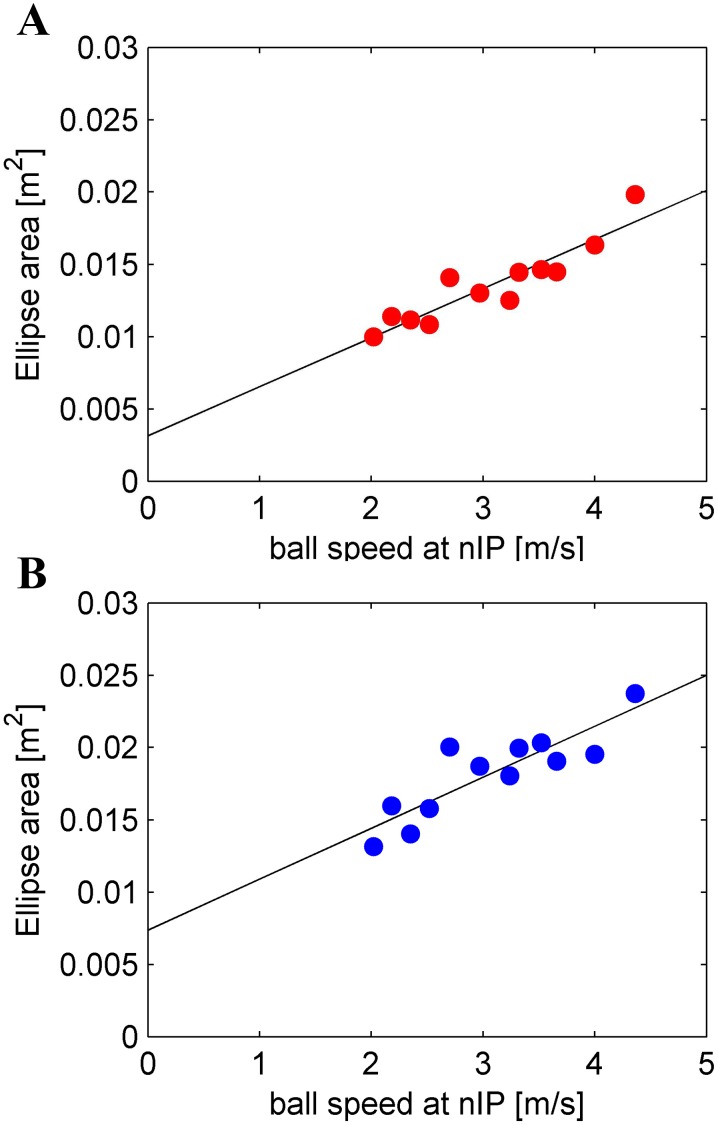
Area of the 95%-tolerance ellipses as a function of ball speed at nIP: data points (red and blue) and linear regression (black). A) Results from Experiment 1. B) Results from Experiment 2.

The area of the ellipses increased significantly (r^2^ = 0.849) with the speed of the ball at nIP (Fig. 6A), indicating that, in different trials, subjects intercepted faster balls at points distributed along a greater path stretch, consistent with the fact that the ball covered a longer path segment in the unit time with higher speeds. The slope of the linear regression was 0.0034 m·s (95% confidence limits: [0.0024, 0.0044]), the intercept was 0.0032 m^2^ (95% confidence limits: [−5·10^−5^, 0.0063]).

**Figure 7:**
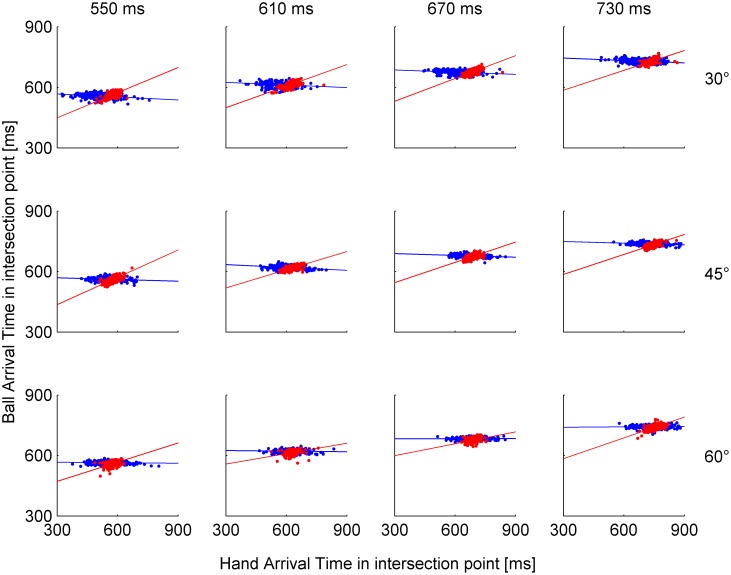
Ball arrival time versus hand arrival time at the intersection point: data points for all single trials (n = 225 for each condition) and linear regression of Experiment 1 (red) and Experiment 2 (blue).

A different way of assessing the subjects’ ability to predict ball trajectory in single trials is provided by the analysis presented in Fig. 7 (red dots). Here we computed the point of the first intersection of the hitter trajectory with the plane of movement of the ball’s center (hitter intersection point), the time at which this occurred (hitter intersection time), the position of the ball when it first reached the minimum distance from the intersection point, and the time at which this occurred (ball intersection time). We then performed a linear regression (red line) between ball intersection time and hitter intersection time. If indeed subjects anticipated ball trajectory, the values of ball intersection time in single trials should covary with the corresponding values of intersection time. This expectation was borne out from the results shown in Fig. 7. For all experimental conditions, the correlation coefficient was significantly higher than zero (P<0.001), the slope of the regression averaged across condition was 0.35, and the average intercept was 0.41. Consistent with the small spatio-temporal errors reported above, we found that the mean contact rate was very high (87.55±1.4%, 3 angles×4 nBMDs×15 subjects, n = 180), implying that subjects were very often successful at hitting the ball close to the nIP (see **Methods**).

#### Movement characteristics

**Figure 8:**
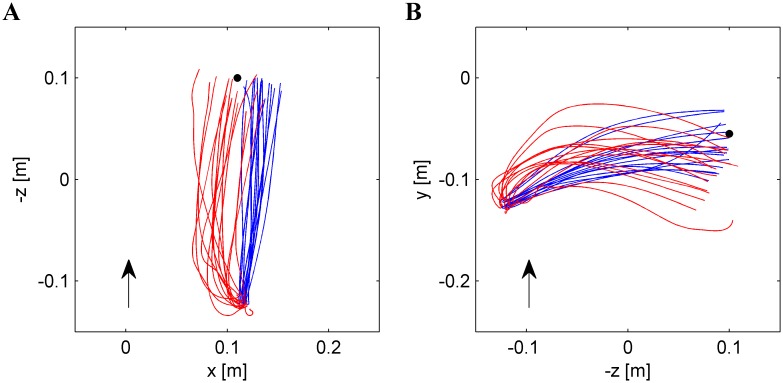
Paths of hand movement in a representative subject who performed both Experiment 1 (red) and Experiment 2 (blue). All repetitions (n = 15, tilt angle = 45°, nBMD = 610 ms) are plotted from the starting position to IP. Black dot represents nIP. 2D projections from above and from right side are plotted in A and B panels, respectively. Arrows indicate forward direction and upward direction of the movement in A and B, respectively.

Typical paths of the hand are plotted in red in Fig. 8, and average movement parameters are presented in Table S4. In general, the hitting movements followed a curved path from an almost invariant starting position (dictated by nSP) to a more variable position close to the interception point. In the previous section we have shown that this spatial variability near interception can be accounted, at least in part, by the attempt of the subjects to intersect the ball trajectory at points distributed along the path of the falling ball.

Path curvature TCurv was quantified in terms of the mean distance of the hitter path from a straight line. Average TCurv was 17.16 mm (SD = 9.01 mm, n = 2640). It depended significantly on nBMDs (3-way RM-ANOVA, 3 angles ×4 nBMDs ×15 repetitions, F_3,42_ = 1.1945, P = 0.011, the shorter the nBMD, the straighter the hitter trajectory), but did not depend significantly on the other factors and their interaction (P>0.085).

A significant dependence on nBMDs was also shown by arm movement duration (MD, F_3,42_ = 3.706, P = 0.019), maximum speed (PSpeed, F_3,42_ = 8.528, P<0.001), and speed at interception (Speed_IP, F_3,42_ = 6.509, P<0.001). Thus, the longer the nBMD, the longer was MD, the higher PSpeed and Speed_IP (see Table S4). Both PSpeed and Speed_IP also depended significantly on repetitions (F_14,196_ = 3.779, P<0.001 and F_14,196_ = 4.549, P<0.001 respectively), increasing with practice. PSpeed depended also on the interaction between nBMD and repetition (F_42, 588_ = 1.6707, P = 0.00603). On the other hand, the initial speed (ISpeed) did not depend significantly on any factor or interaction (P>0.127). The observation that ball motions of higher final speed (longer nBMD) were associated with faster arm movements agrees with several previous reports on the interception of targets with variable speeds under visual guidance [11], [53], [54].

**Figure 9:**
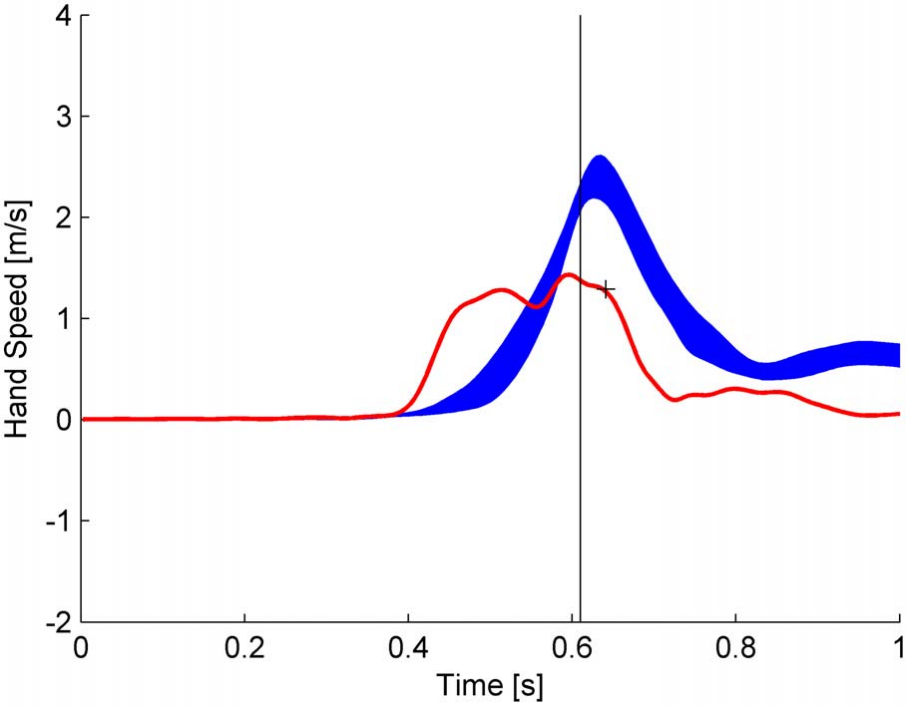
Experiment 1. Speed profiles of hand movement of a representative subject (different from that of Fig. 8). The profile of the first repetition (red) is superimposed on the average (±95% confidence interval, blue) over 4^th^ to 15^th^ repetitions of the same condition (tilt angle 45°, nBMD = 610 ms). Traces are aligned on the start time of ball motion. nBMD is marked by the vertical line, and the hand arrival time at IP in the 1^st^ repetition by the cross.

Typical speed profiles are plotted in Fig. 9 for a representative subject. It can be noticed that the profile for the first repetition (red) does not overlap with the average profile (blue) computed over the repetitions from 4th to 15th of the same condition, but is anticipated in time. In addition, while the average profile exhibits the typical bell-shaped waveform of fast reaching movements with little, if any, on-line corrections [55], the profile of the first repetition is biphasic. Hand speed reached a first peak and then decreased about 100 ms prior to the nominal interception time, to peak again around interception time. This biphasic profile was observed in the very first trial of all subjects, and more sporadically in the first repetition of the other conditions. When the profile of the first repetition was not obviously biphasic, the rise time of speed was nevertheless slower than in the following repetitions. The presence of submovements in the tangential speed profile may be indicative of on-line corrections, possibly based on visual feedback.

In sum, the average interception position and time were very close to those required by the instructions, and this was so at all tilt angles and starting positions of the ball on the incline. Individual interception positions in single trials tended to be distributed along the trajectory of ball motion, consistent with the hypothesis that subjects were able to fully extrapolate the trajectory. There was also clear evidence for rapid learning: timing errors were reduced and movements became more stereotyped after the first few repetitions of practice. Also, balls arriving at higher speed were intercepted more vigorously, with faster movements. A similar covariation between arm speed and target speed has previously been shown to result from continuous adjustment of interceptive movements based on visual feedback of target speed with a delay of about 200 ms [11].

The findings from Experiment 1 are compatible with the hypothesis that participants took into account both the translational and rotational component of rolling motion of the ball on the incline, as well as the subsequent free-fall kinematics. As a result, they were able to deal with widely different accelerations on the incline and final speeds at interception over the full range of conditions.

## Experiment 2

The previous experiment showed that, with continuous visual feedback plus haptic feedback of hand-ball contact in successful trials, subjects are able to extrapolate accurately the gravitational motion of a real target with very different kinematics. We now address the question of whether extrapolation is still possible when there is no visual feedback about target kinematics during the critical terminal part of the trajectory, nor haptic feedback of hand-ball contact.

In Experiment 2, the ball rolled along the incline as in Experiment 1, but it was stopped just before the end of the track. Participants were asked to imagine that the ball kept moving, and to punch it with the hitter at the same nominal interception point as in Experiment 1. It should be remarked that, because the unseen portion of ball trajectories mainly corresponded to a free-fall along quasi-parabolic paths, a simple linear extrapolation of the visible portion would not allow a correct prediction of the interception point and time, at least for 30° and 45° tilts (see Fig. 5).

### Methods

#### Participants

Fifteen subjects (11 females and 4 males, 28±7 years old) participated in Experiment 2. Seven of them had previously participated in Experiment 1 (about 5 months before).

#### General procedures

The experimental setup, task and protocol were similar to those of Experiment 1, with the following notable changes. In this experiment, the ball rolled along the incline until it was stopped by a rod placed 2 cm before the end of the track, and remained there for the rest of the trial. Participants were asked to imagine that the ball kept moving beyond the stop. They had to punch the imaginary ball with the hitter at the same nominal interception point (nIP) as in Experiment 1. Notice that they never saw the ball falling off the incline during this experiment.

Data analysis was identical to that of Experiment 1, except that contact rate could not be assessed, given that there was no physical interaction with the ball. Out of a total of 2700 trials (180 trials×15 subjects), 39 were excluded (1.4%) from the analysis due to the presence of artifacts or lack of subject’s attention. Because we did not find any systematic difference between the group of 7 subjects who had previously performed Experiment 1 and the group of 8 subjects who did not, the results from the two groups were pooled together.

### Results and Discussion

#### Interception performance

Despite the virtual nature of the interception which lacked visual and haptic feedback of a hit ball, both the temporal and spatial errors were limited. The mean timing error TE (computed over all repetitions of all conditions) was −13 ms (SD = 69 ms, n = 2661, Fig. 2B), within the theoretical margin of error. However, there was a gradient of the response timing with increasing tilt angle: on average, TE was −51±67 ms, 3±57 ms and 15±68 ms for 30°, 45° and 60°, respectively. Three-way RM-ANOVA (3 angles×4 nBMDs×15 repetitions) showed that TE depended significantly on angle (F_2,28_ = 25.76, P<0.001), whereas it was independent of nBMD, repetition and any interactions (P>0.062). Post-hoc tests showed that TE for 30° was significantly different from that for the other angles (P<0.001).

This trend is not compatible with the possibility that subjects extrapolated the visible trajectories under the assumption that the ball continued rolling on an extended inclined plane. If this were the case, in fact, the responses should be late (because rolling is slower than free-fall), with the longest time delay for 30° tilt and smaller delays with increasing tilt angle, the opposite trend of what we found.

A possible explanation for the early responses at 30° tilt is that subjects misperceived this tilt angle. It is known that observers tend to overestimate the slant of an incline [42], [56], more so for smaller than larger tilts [57]. Such a differential estimate of tilt angle might account for the observed gradient of responses, because larger overestimates of 30° tilt would predict earlier responses than for 45° or 60° tilts. However, it has been shown that the estimates of an incline tilt are generally correct when an action is performed on the incline [42], [57].

A more plausible explanation is that the gradient of responses as a function of tilt angle is related to a central tendency effect. The ball motion that subjects had to imagine in order to fill in the occluded gap (that is, the time gap between ball stop on the incline and the theoretical ball arrival at nIP) lasted less and less with increasing tilt angle (see Table S1). If subjects assumed that the occluded duration of fall was the same for all angles and equal to that for 45° angle, they would arrive at nIP early for 30°, on time for 45°, and late for 60°. Indeed, the difference between mean TE for 30° and that for 45° (54 ms) roughly corresponds to the mean difference in duration of fall for these two angles (38 ms), and the difference between mean TE for 45° and 60° (12 ms) roughly matches the corresponding difference in duration of fall (15 ms). This observation suggests that subjects used an interceptive strategy based on a global assessment of the unseen ball kinematics across all tested conditions. In this regards, it should be recalled that tilt angle was blocked (although in counterbalanced order across subjects), while nBMD was randomized across trials.

The overall mean spatial error (distance between IPs and nIP) was 4 cm (SD = 2 cm, n = 2661), essentially identical to that of Experiment 1. Spatial error (Fig. 4B) depended slightly but significantly on tilt angle (3-way RM-ANOVA, 3 angles×4 nBMDs×15 repetitions (F_2,28_ = 6.54, P<0.005) and repetitions (F_14,196_ = 3.997, P<0.001), while it was independent of nBMD and any interactions (P>0.178). Post-hoc tests revealed that the spatial error was significantly smaller at 30° than 60° angle (P<0.005), while it was significantly greater in the first repetition than all other repetitions (except repetitions 2 and 5).

**Figure 10:**
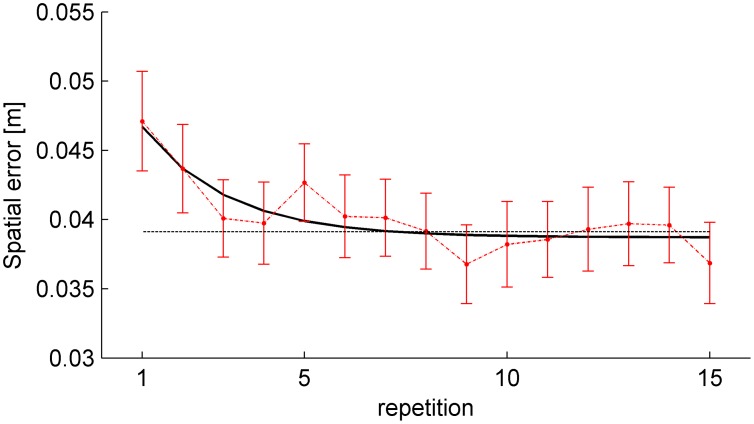
Experiment 2. Effect of practice on spatial errors. Mean error (±95% confidence interval, over all subjects, tilt angles and nBMD, n = 180) is plotted for each repetition (red), together with the exponential best-fit (black), and the 95% decrement (dotted gray).

The effect of practice on the spatial error was quantified by best-fitting (r^2^ = 0.721) eq. 1 to the spatial error of all subjects as a function of repetition (Fig. 10). We found a short learning constant (*b_2_* = 2.095, 95% confidence limits: [0.4363, 3.753]), so that the error was reduced by 95% of the overall change relative to the steady state value after 6 repetitions. The other fitting parameters were: offset *b_0_* = 0.03871 m (95% confidence limits: [0.03753, 0.0399] m) and gain *b_1_* = 0.008 m (95% confidence limits: [0.005095, 0.0109] m).

**Figure 11:**
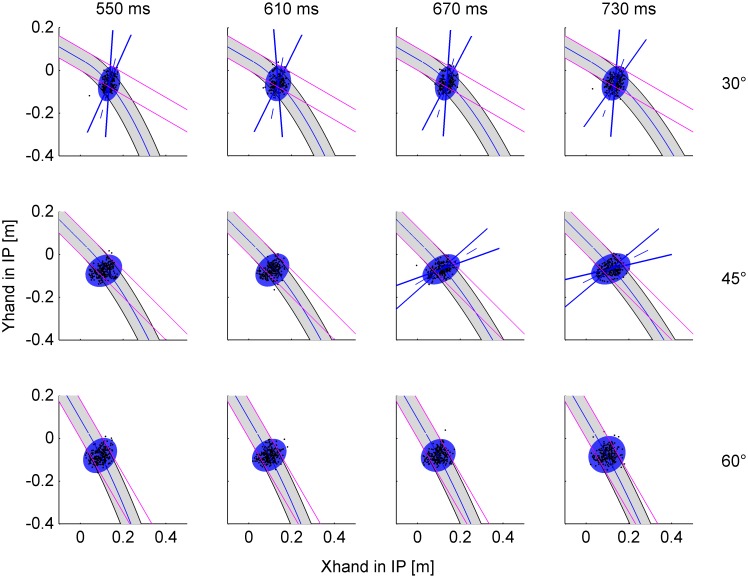
Experiment 2. Spatial distribution of interception points. IPs in single trials (black dots) and 95%-tolerance ellipses (blue) for each condition. The first eigenvector with the 95%-confidence cone is drawn when significant. Same format as in Fig. 5.

Although the errors were limited, in contrast with Experiment 1 the spatial scatter of individual reaches was not correlated with the virtual path of the ball from the incline until the nominal interception point. Figure 11 shows the tolerance ellipses including 95% of the interception points IPs (T*_0.95_*) for each experimental condition (n = 12, 4 nBMD×3 angles). We found that only 6 over 12 ellipses had statistically different eigenvalues (Table S5). Moreover, the 95% confidence limits of the first eigenvector never included the tangent in the mean IP to the trajectory of the ball, deviating from it by >50° (Table S6). Therefore, the individual responses were scattered randomly around the nIP, consistent with the hypothesis that subjects were unable to fully predict the invisible trajectory.

Nevertheless, subjects did take into account the theoretical speed of the ball at arrival. In fact, the area of the ellipses increased significantly (r^2^ = 0.848) with the speed of the ball at nIP (Fig. 6B), similarly to Experiment 1. The slope of the linear regression was 0.0035 m·s (95% confidence limits: [0.0024, 0.0044]), the intercept was 0.0074 m^2^ (95% confidence limits: [0.0029, 0.0118]).

In contrast with Experiment 1, hitter intersection time was not significantly correlated with ball intersection time (Fig. 7). For no experimental condition was the correlation coefficient significantly higher than zero (P>0.21); the slope of the regression averaged across condition was −0.0257, and the average intercept was 0.66. Therefore, if one considers the spatial distributions of IPs and the lack of correlation between IT and ball intersection time distributions, it appears that the *where* and *when* subjects placed their hand to intersect ball trajectory were poorly related to each other.

#### Movement characteristics

Typical paths of the hand are plotted in blue in Fig. 8, and average movement parameters are presented in Table S7. Hitting movements were systematically straighter and of shorter duration than those in Experiment 1 (red traces in Fig. 8). Average path curvature TCurv was 9.06 mm (SD = 4.71 mm, n = 2661), significantly smaller than in Experiment 1 (F_1,28_ = 20.307, P = 0.00011). Average movement duration MD was 227.49 ms (SD = 61.24 ms), significantly shorter than in Experiment 1 (F_1,28_ = 12.05, P<0.005). None of the movement parameters depended significantly on any experimental factor (3-way RM-ANOVA, 3 angles×4 nBMDs×15 repetitions, P>0.072), except for the peak speed (PSpeed) and the speed in IP (Speed_IP) which depended on nBMD (F_3,42_ = 6.183 and F_3,42_ = 14.51, both P<0.001), being higher for longer nBMDs (higher target speeds).

In sum, the average interception position and time were roughly close to those required by the instructions. Subjects tended to use an interceptive strategy based on a global assessment of the unseen ball kinematics across all tested conditions. A fine tuning of the responses to the ball kinematics of single trials was limited, due to the lack of on-line feedback about the critical terminal portion of ball trajectory. In contrast with Experiment 1, the individual responses were scattered randomly around the nIP, and hitter intersection time was not significantly correlated with ball intersection time. Nevertheless, there were indications of some tuning of individual responses: the area of the 95%-tolerance ellipses of the interception points increased significantly with the speed of the ball at nIP, and the speed of arm movement was significantly greater for faster balls than for slower balls, as in Experiment 1. Learning involved spatial but not temporal parameters of interception, presumably because participants received feedback about the former but not the latter.

Overall, the results showed that subjects can still extrapolate global aspects of gravitational motion of a target falling from an incline even when the target stops moving before the fall, so that there is no visual feedback about target kinematics during the free-fall and no haptic feedback of hand-ball contact. In this experiment, subjects were required to predict the expected time of arrival of the ball at the nominal interception point. In the next experiment, we tested whether subjects are also able to predict the full trajectory of free-fall after the descent along the incline.

## Experiment 3

In this experiment, the ball rolled along the incline until it was stopped near the end of the track, as in Experiment 2, but here participants were asked to draw with the hand in air the imaginary trajectory of the ball, trying to mimic its motion beyond the stop. They never saw the ball falling down the experimental incline during or prior the experiment, nor were they told anything about its possible motion. Mental extrapolation of the ball trajectory could be based only on visual information obtained from the previous rolling motion along the incline and on an internal model of free-fall.

### Methods

#### Participants

Twelve subjects (9 females and 3 males, 23±1 years old) participated in the experiment. None of them had served in the previous experiments.

#### General procedures

The experimental setup and protocol were similar to those of Experiment 2. In different blocks of trials, the incline was tilted by 30°, 45° or 60° relative to the horizontal (counterbalanced order). At each tilt angle, the ball was randomly released from one of 4 different positions, resulting in different ball motion durations nBMD (Table S8). These positions had been chosen so that at least two of them for each angle were the same as in Experiment 1 and 2, one was the same for all angles, and one resulted in nearly the same ball speed (2.7 m·s^−1^) at the lower end of the incline for all angles.

As in Experiment 2, the ball rolled along the incline until it was stopped by the rod near the end of the track. In the present experiment, participants waited with the adducted, semi-flexed upper limb, mid-pronated wrist. In this specific starting position, the tip of the hand-held hitter rested at the stop-rod. Upon arrival of the ball at the stop, participants had to immediately start tracing with the hitter in air the imaginary trajectory of the ball, as if it kept moving beyond the stop.

#### Data analysis

The kinematics of the hand-held hitter was preprocessed as in the previous experiments. Next, we analyzed both spatial and temporal aspects of the hand movements.

*Spatial analysis*. To compare trials with variable length of hand movements, we considered only the portion of each trajectory from the starting position to a final position at 12-cm Euclidean distance from the starting position (all movements spanned at least this distance), roughly comparable to the interception point in Experiments 1 and 2. The trajectories were discretized in 100 equidistant points between starting position and final position. The mean trajectory, along with the 95% confidence interval, was computed over all trials for each condition (tilt angle and nBMD). The same discretization was applied to the actual ball trajectory during free-fall from the incline, as measured in separate calibration trials (see **Appendix**). The deviation (TDe) of the hand trajectory in single trials from the ball trajectory was computed as the root mean square of the distances between each pair of corresponding points on the two discretized trajectories. The main direction TD of hand trajectory was defined as the slope of the starting position-final position segment, and was compared with the direction of actual ball trajectory defined in the same manner.

*Temporal analysis*. In contrast with the relatively stereotyped hand movements of the two previous experiments, movements were more variable in this experiment. Thus, for each trial, the time of onset of hand movements was derived according to an algorithm recommended by Teasdale et al. [58] and Tresilian et al. [59]. The algorithm involves the following steps: a) the tangential velocity (*v*_T_) is normalized to the maximum *v*_max_: *v* =  *v*_T_/*v*_max_; b) the sample k at which *v* first exceeds 0.1 is located; c) going back from sample k, one stops at the first sample (m) for which *v*≤0.09; d) the standard deviation SD_v_ of *v* is computed between sample m and sample k; e) going back from sample m, one stops at the first sample for which *v* ≤ *v*_m_ – SD_v_: this is the onset time (OT).

#### Statistics

Kolmogorov-Smirnov test showed that the trajectory directions and deviations were not normally distributed (P<0.001). Accordingly, for each tilt angle, the dependency of the trajectory parameters on nBMD was assessed using Kruskal-Wallis nonparametric test. Whenever a parameter did not depend significantly on nBMD, statistics on its median and dispersion were performed using Kruskal-Wallis and Ansari-Bradley tests with tilt angle as factor (with Bonferroni correction). Statistics on the characteristics of hand movements were performed using *t*-statistics separately for each tilt angle and nBMD. Post-hoc Bonferroni corrections were applied (P<0.05).

### Results and Discussion

**Figure 12:**
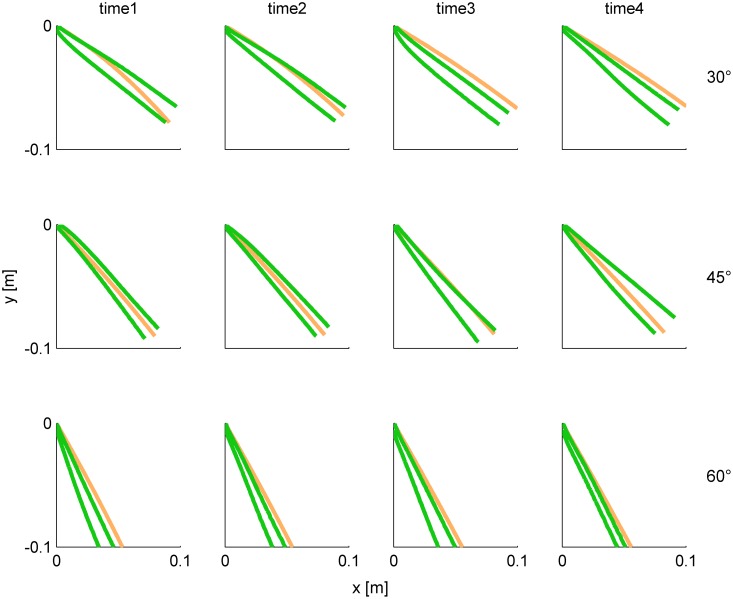
Experiment 3. Frontal plane projection of 95%-confidence limits of hand paths (green, over all repetitions, n = 15) and actual ball path (orange) for each condition in a representative subject.

Although the participants never saw the ball falling off the incline, on average they were able to draw its trajectory in air reasonably well. The 95% confidence limits of the hand paths computed over all repetitions of a representative subject are compared with the actual ball trajectories for each condition in Fig. 12. In half of the conditions (6/12), the confidence limits included the actual ball trajectory, while in the other half the confidence limits did not include the ball trajectory but the discrepancy was limited.

**Figure 13:**
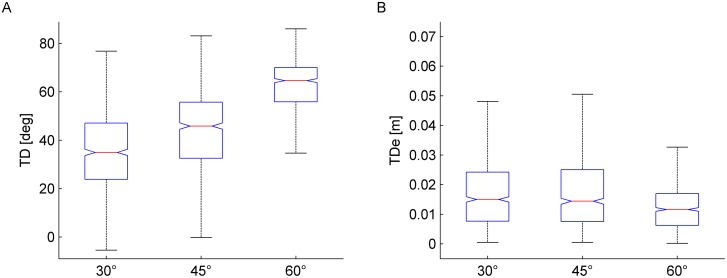
Experiment 3. Box-and-whisker plots of main direction TD of hand trajectory (A) and root mean square deviation TDe from the actual ball trajectory (B). Bottom and top of the boxes correspond to the lower and upper quartile, respectively, and define the IQR. The intermediate line in each box is the median. The lower and upper ends of the whiskers correspond to the smallest and largest datum within 1.5 IQR. Results from all nBMD, repetitions and subjects have been pooled for each condition (n = 720).

Summary results from all participants are shown in Fig. 13. At each tilt angle, the main direction TD of hand trajectory did not depend significantly on nBMDs (all P>0.19). Instead, TD depended significantly on tilt angle (P<0.001): the greater the angle, the greater the TD. Median values of TD (n = 720 = 4 nBMD×15 repetitions×12 subjects) were 34.97°, 45.87° and 64.68° for 30°, 45° and 60°, respectively (Fig. 13A). Importantly, these median values of TD were not significantly different from the corresponding directions of actual ball trajectory (Kruskal-Wallis test, P>0.5), which were 36.13°, 47.65°, and 61.40° for 30°, 45° and 60°, respectively. This result confirms that, on average, subjects were able to draw the trajectory in the correct direction. However, as shown by Fig. 13A, there was considerable inter-trial variability.

As a further test of the drawing accuracy, we derived a global error estimate computed as the root mean square deviation (TDe) of the discretized trajectory of the hand relative to the actual ball trajectory (Fig. 13B). We found that, at each tilt angle, TDe was small and did not depend significantly on nBMD (all P>0.40). Instead, TDe depended on tilt angle (P<0.001). The greater the angle, the smaller was the TDe: the median TDe was 1.46, 1.40 and 1.03 cm for 30°, 45° and 60°, respectively (the differences between 60° and 30° and between 60° and 45° were significant with Bonferroni test). Also the dispersion of TDe around the median depended significantly on tilt angle (Ansari-Bradley test), but did not depend on nBMD. The greater the angle, the smaller was the dispersion of TDe (the differences between 60° and 30° and between 60° and 45° were significant after Bonferroni test).

#### Temporal movement characteristics

Average movement parameters are presented in Table S9. For each condition, the onset of hand movements (averaged over all subjects and repetitions of each condition) occurred earlier than the arrival of the ball at the stop (paired t-test, P<0.001), while the peak speed occurred later than ball arrival at the stop (paired t-test, P<0.001). The peak speed increased significantly (least-squares linear regression, r^2^ = 0.601, n = 180) with the speed of the ball at the lower end of the incline. The slope of the linear regression across all conditions was 0.2609 (95% confidence limits: [0.2294, 0.2924]), and the intercept was 1.271 m·s^−1^ (95% confidence limits: [1.177, 1.366]). Although the peak speed of hand movements did not match that of actual ball motions, it was substantially more modulated across conditions than in the previous experiments. Thus, while the difference between maximum and minimum peak speed measured in different conditions was 6.5 and 6.2% of the minimum value in Experiment 1 and 2, respectively, the same difference was 41.6% in Experiment 3 (cf. Tables S4, S7, S9).

In sum, subjects were able to mimic with hand movements several features of unseen free-falls of the ball across a range of different conditions. Average hand movements reproduced to a good extent the path and overall direction of the actual ball trajectory. Moreover, hand movements were performed with a wide range of peak speeds with a trend similar to the range of ball speeds across conditions. Subjects were more precise (less variable) when they drew ball trajectories corresponding to greater tilt angles.

## General Discussion

We found evidence that participants took into account the physics of fall from an inclined plane in all three experiments. Not surprisingly, performance was most accurate when motion of the ball was visible until interception and haptic feedback of hand-ball contact was available (Experiment 1). Nevertheless, even when ball motion was stopped at the end of the incline and participants punched an imaginary moving ball (Experiment 2) or drew in air the imaginary trajectory (Experiment 3), they were able to extrapolate to some extent global aspects of the target motion, including its path, speed and arrival time.

In Experiment 1, the ball underwent two different laws of motion in successive phases of the descent. It first rolled down the incline with a linear, roto-translatory motion whose kinematics depended on the tilt angle and starting position. Afterwards, the ball fell freely along a quasi-parabolic path which depended on the exit velocity (modulus and direction) from the incline. The deviation of the free-fall path relative to the previous rectilinear path was appreciable for 30° and 45° tilts, but negligible for 60° tilt. Ball acceleration was a fraction of 1*g* during the rolling phase, whereas it was 1*g* during the free-fall phase at all tilts. Therefore, there was no simple linear relationship between target motion during free-fall and the previous rolling phase of the trajectory. Nevertheless, the results showed that target motion was fully extrapolated by the participants. At all tilt angles and starting positions of the ball on the incline, the mean interception point and time were very close to the ideal values, and the individual movements in single trials tended to intersect the plane of ball motion at points scattered along the path of fall (Fig. 5).

In this experiment, punching movements were most likely under on-line visual control, as suggested by several indirect clues: the distribution of interception positions along the ball path mentioned above, the presence of significant curvature in hand movements in the direction of target motion (Fig. 8), the robust coupling between hand speed and target speed and an appreciable movement duration (Table S4). Moreover, submovements were recognizable in the tangential speed profile of the initial repetitions (Fig. 9). All such behavioral hallmarks have previously been interpreted as related to corrective interventions, mainly based on visual feedback (see [11], [60]–[64]).

Visual information, however, could not be used for on-line adjustments of hand movements during the free-fall phase preceding the interception, because the exit time of the ball from the incline always occurred within the presumed “blind” period corresponding to the visuomotor delay (e.g., [12], [26], [60]). In fact, it is known that typical visuomotor delays for manual interceptions range between 100 and 300 ms (e.g., [3], [11]–[13]), whereas the duration of free-fall from the exit time until nominal interception time was only 18 to 80 ms, depending on the condition (see Table S1). However, visual information and haptic feedback at ball-hand contact (or the lack of it with a missed interception) could be used to update the planning of movement for the next trials [65].

Extrapolation of target motion over the free-fall phase was presumably based on the combination of multiple cues: visual information obtained over the preceding rolling phase, feedback and memory from previous trials, and an internal model of physics. The combination of these cues resulted in an accurate estimate of ball descent. The involvement of on-line visual information during rolling has already been discussed. A memory representation of target motion could be built from initial trials using visual and haptic feedback as discussed above. Consistent with this hypothesis, we found a rapid improvement of interception timing (Fig. 3). However, rote memory alone was probably insufficient to ensure a full representation of target motion during free-fall, because the initial conditions of ball motion were randomized across trials and therefore the exit velocity from the incline was unpredictable. In particular, while tilt angle was blocked (in counterbalanced order), the duration of ball motion (nBMD) was randomized across trials.

We suggest that an internal model of target dynamics played a key role in motion extrapolation. This putative internal model is considerably more complex than that involved in the interception of a target falling vertically with constant acceleration [8], [23], [25], because ball motion in the present experiments depended on several physical parameters, some invariant across trials (such as the ball and surface properties, air drag), and others variable across trials (such as the surface inclination and starting position on the incline). A somewhat similar internal model, able to account for complex acceleration profiles, has previously been suggested by De Rugy et al. [44].

We did not monitor eye movements in our experiments, but a previous study showed that naturalistic rolling objects elicit effective tracking eye movements [43]. Thus, one may speculate that also in our experiments the internal model of target motion guided anticipatory eye movements along the expected trajectory, and in turn the efference copy of eye movements guided the hand to intercept the target at the expected position and time.

The potential role of the internal model of target motion was even more crucial in Experiments 2 and 3, where the participants saw the rolling phase of ball motion along the incline but did not have any visual or haptic feedback about target kinematics during the critical terminal part of the trajectory. Indeed, it has previously been shown that the role of the internal model of gravity effects becomes prevalent in the presence of visual occlusions over the terminal part of a motion trajectory [66]–[68].

In Experiment 2, the average interception position and time were close to those required by the instructions, but the individual responses were tuned to the specific conditions of single trials to only a limited extent. Indeed, there was no clear directional distribution of the individual interception positions along the ball path (Fig. 11). The observation that, in comparison with the hitting movements of Experiment 1, those of Experiment 2 started later, lasted less, were straighter, and did not exhibit obvious on-line corrections speaks in favor of the idea that the latter movements were more stereotyped and relied less on visual on-line information. The bulk of the data from Experiment 2 suggests that subjects used an interceptive strategy based on a global assessment of the unseen ball kinematics across a range of different conditions, while exploiting the residual visual information to maintain some degree of hand-target motion coupling (as shown by the covariation of the speed of arm movement with that of the falling ball). This interpretation is in keeping with the previous suggestion that, in the presence of visual occlusions over the terminal part of a motion trajectory, subjects develop a new interception strategy specifically adapted to the occluded protocol [68], [69].

In Experiment 3, the subjects were able to draw in air hand trajectories which roughly reproduced several features of the actual ball trajectories across a range of different conditions, such as ball path, direction and speed. Much previous work investigated the nature of the internal representations of dynamics that are associated with the observation of an object in motion (e.g., [18], [21], [40], [48], [70]–[72]). These representations vary widely depending on the context, yielding behavioral responses that are compatible with naïve physics, geometric kinematics, simple heuristics, or Newtonian physics (for a recent account, see [18]). In general, when the visual presentation of the target is animated (as opposed to a static picture) and the response required by the observer is a motor action (as opposed to an explicit judgment or verbal response), as in the case of our experiments, the responses tend to be consistent with internalized Newtonian physics, although the internal representations often model actual events only in an approximate manner [21], [26].

In conclusion, all 3 experiments showed that ball path and kinematics generated by complex force patterns can be extrapolated surprisingly well by the brain using both visual information and internal models of target motion.

## Appendix

Here we report the parameters of ball motion as derived from a series of calibration trials performed before the experiments.

### Characterization of Ball Motion

#### Equations of motion on the inclined plane

The equation of motion of a homogenous sphere rolling without slipping on a tilted surface, under gravity and air drag linear in speed, is (see Fig. S1 and Fig. S2B):
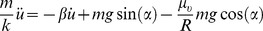
(A1)where 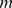 is the mass of the sphere, 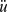 is the linear acceleration of its center of mass along the incline axis, 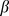 is the viscous friction coefficient (it takes into account air viscosity, size and shape of the ball moving through the air), 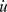 is the velocity of its center of mass along the incline axis, *g* is the gravitational acceleration (9.81 m s^−2^), 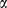 is the angle of tilt of the incline relative to the horizontal, 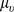 is the coefficient of rolling resistance (unit of length), 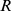 is ball radius.

The moment of inertia 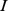 of a sphere with the axis through the center of mass is
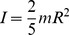
(A2)therefore
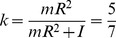
(A3)With
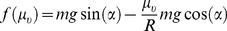
(A4)Equation A1 then reduces to



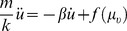
(A5)If we integrate Equation A5 with initial conditions 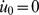 and 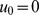, we obtain velocity:
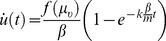
(A6)and position:
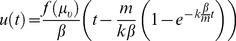
(A7)


#### Characterization of the incline

Given the above expressions for speed and position (Eq. A6 and A7), a full characterization of ball motion on the incline can be achieved once 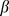 and 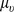 are known. 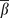 and 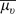 were evaluated with a procedure based on the analysis of digital images of ball trajectories recorded at 100 Hz by a Digital Video Camera (Sony Handycam HDR-SR8E). These parameters were estimated experimentally by analyzing 36 video recordings of ball rolling motion (spatial resolution: 1920×1080 pixels, temporal resolution: 100 interlaced fields per second) on the incline (between the releasing position and the lower end of the incline) inclined at 45°. We performed 3 video recordings for each of the 12 releasing positions of Experiment 1 and 2. The beginning of video recording and that of ball motion were not synchronous. Therefore, we identified the onset frame of video-recorded ball motion as the first video frame at which the ball center of mass exceeded the threshold velocity of 0.1 m·s^−1^. The ball position in this frame is indicated as *u*_0_, and ball speed as V_0_.

Motion equation for video recorded ball motion was obtained integrating twice Equation A5 with initial conditions 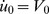 and 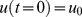:
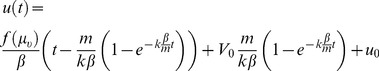
(A8)


The parameters 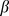 and 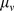 were obtained by (nonlinear least-squares) best-fitting Equation A8 to the ball kinematics recorded in the videos from the onset frame of video-recorded ball motion and the end of motion recording. The estimated value of 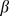 was 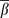  = 1.77·10^−5^ (±1.79 10^−5^ SD, n = 36) and the estimated value of 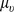 was 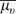 = 2.2 10^−3^ (±5.17·10^−4^ SD).

Ball acceleration was roughly constant on the plane for a given inclination. It was 3.21 (±0.25·10^−3^ SD) m·s^−2^, 4.71 (±0.39·10^−3^ SD) m·s^−2^ and 5.90 (±0.50·10^−3^ SD) m·s^−2^ for tilt angles of to 30°, 45° and 60°, respectively (mean and SD was computed for the longest ball motion duration on the plane with 1 ms time step).

#### Ball motion in air

The equation of motion of a sphere in air (i.e. after exiting from the lower end of the incline), under gravity and air drag linear in speed, is (see Fig. S2C):
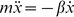
(A9)

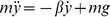
(A10)where 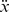 (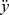) is the linear acceleration of its center of mass along the horizontal (vertical) axis and 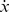 (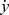) is the velocity of its center of mass along the horizontal (vertical) axis (Fig. S1).

The corresponding velocity profiles are:
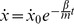
(A11)

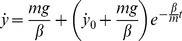
(A12)and positions are:



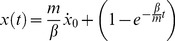
(A13)

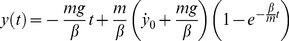
(A14)


Solutions were obtained by integrating Equation A9 and A10 with initial conditions, 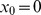, 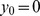 and 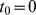. In the above equations, the point 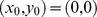 represents the position of the center of mass of the ball when it exits from the incline.

We verified that edge effects were negligible in the experimental conditions by means of measurements of the ball speed at the lower end of the incline and of the flight time of the ball in air through the high resolution video-camera and the 3-axial accelerometer placed at 1 ball-radius distance below and to the right from the nominal interception point.

### Comparison of Different Laws of Motion

Figure S2 (right panels) plots the isochronous lines corresponding to falls under different conditions. Different colors identify different durations of fall (see Figure legend). Each isochronous line depicts the locus of starting positions yielding constant duration of fall along planes of different inclinations. For sliding motion under gravity on a friction-less plane, the isochronous lines are circles passing through the end of the planes (Fig. S2A). For rolling motion without slipping under gravity and air drag linear in speed (Eq. A1) and for free-fall after rolling on the incline, the isochronous lines become egg-like (Fig. S2B–C).

## Supporting Information

Figure S1 **Schematic of ball motion.** The ball is illustrated in 3 different positions: while rolling down the incline, at the exit of the incline, and at the nominal interception point (at distance d_1_ from the incline exit).
(TIF)

Figure S2 **Schematic illustration of different types of falls from an inclined plane.** A. Sliding of a parallelepiped under gravity on a friction-less plane. B. Rolling without slipping of a sphere under gravity and air drag linear in speed. C. As in B, followed by free-fall under gravity and air drag. Left panels: free-body diagrams of falls. *α*: inclination angle relative to the horizontal. F_N_: ground reaction force. F_g_: gravitational force. F_β_: air resistance force. F_s_: sliding resistance. M_v_: rolling resistance moment. In C, d_1_ corresponds to the arrival point after free-fall (as in Fig. S1). Right panels: isochronous lines for falls at different tilt angles. Each isoline connects the x,y starting positions yielding the same duration of fall along planes of different inclinations: red for 550 ms, cyan for 610 ms, green for 670 ms, and purple for 730 ms. In A, the isolines corresponding to 670 and 730 ms are not drawn because out-of-scale for higher tilt angles. In C, the isolines have been computed for d_1_ = 0.15 m.
(TIF)

Table S1 **Ball motion parameters in Experiment 1 (Incline and Air) and Experiment 2 (Incline).**
(DOCX)

Table S2 **Square-root of the eigenvalues of 95% tolerance ellipses in Experiment 1.** They correspond to the semi-axes of the ellipses (cm). *Eigenvalues not statistically distinct.
(DOCX)

Table S3 **Orientation (in degrees) of the major axis of 95% tolerance ellipses in Experiment 1.**
(DOCX)

Table S4 **Mean values and standard deviations (SD) of the kinematical variables MD, ISpeed, PSpeed, Speed_IP and TCurv as a function of the four nBMDs and the three incline tilting angles in Experiment 1.**
(DOCX)

Table S5 **Square-root of the eigenvalues of 95% tolerance ellipses in Experiment 2.** They correspond to the semi-axes of the ellipses (cm). *Eigenvalues not statistically distinct.
(DOCX)

Table S6 **Inclination (in degree) of the major axis of 95% tolerance ellipses in Experiment 2.**
(DOCX)

Table S7 **Mean values and standard deviations (SD) of the kinematical variables MD, ISpeed, PSpeed, Speed_IP and TCurv as a function of the four nBMDs and the three incline tilting angles in Experiment 2.**
(DOCX)

Table S8 **Ball motion parameters in Experiment 3.**
(DOCX)

Table S9 **Experiment 3.** Mean values and standard deviations (SD) of the kinematical variables: time interval between OT and ball stop time (BST), PSpeed, Interval time between PSpeed time and BST as a function of the four ball motion duration for each incline tilt and the three incline tilting angles. Note that ball motion duration time1, time2, time3, time4 were not the same for the three incline tilting angles.
(DOCX)
