# Supplementary figures and images for: Neural Extrapolation of Motion for a Ball Rolling Down an Inclined Plane
Source: PLoS One. 2014 Jun 18;9(6):e99837. doi: 10.1371/journal.pone.0099837 (PMC4062474; doi:10.1371/journal.pone.0099837)

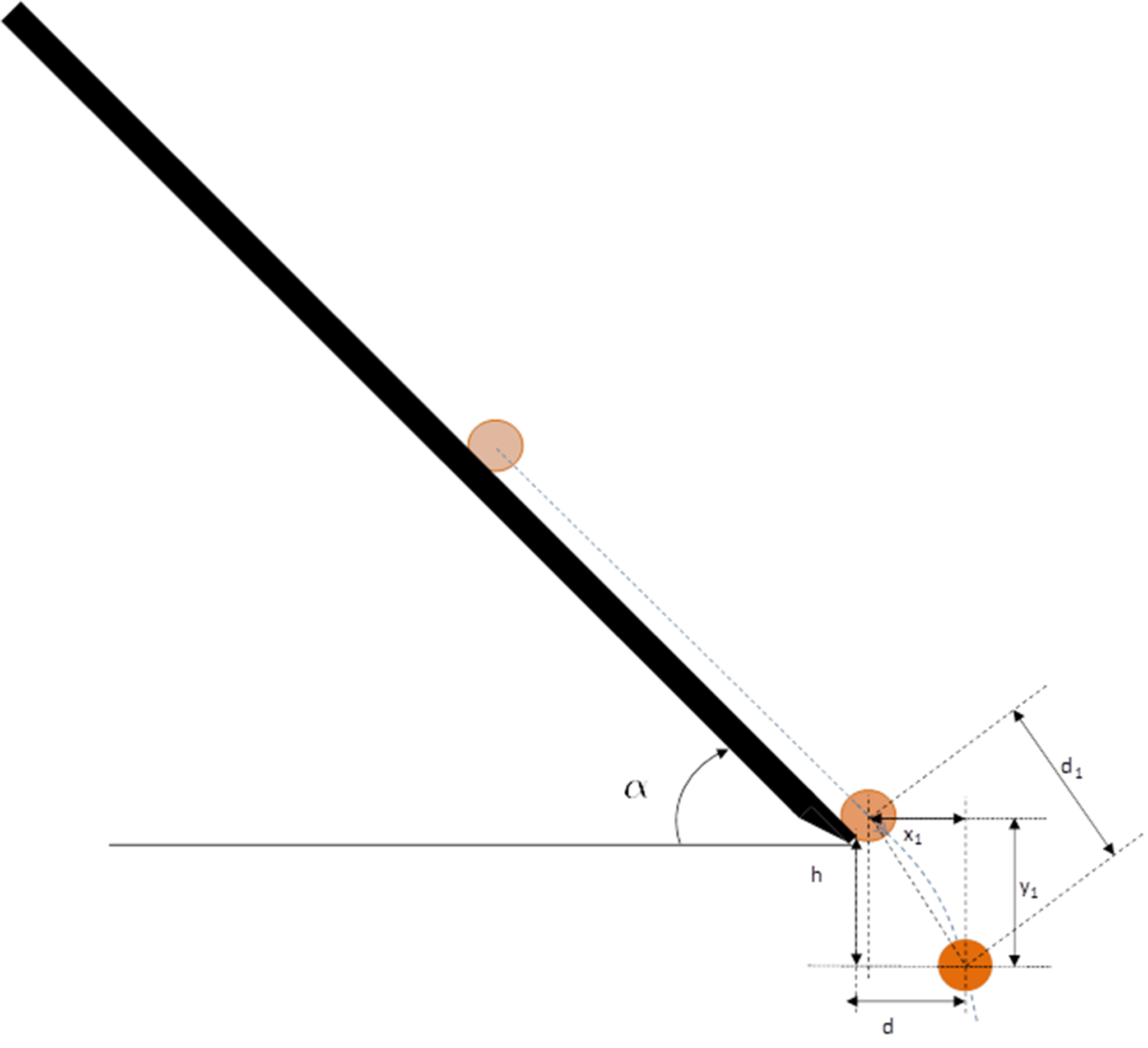

Supplement: Figure S1 — Schematic of ball motion. The ball is illustrated in 3 different positions: while rolling down the incline, at the exit of the incline, and at the nominal interception point (at distance d1 from the incline exit). (TIF) [file pone.0099837.s001.tif]

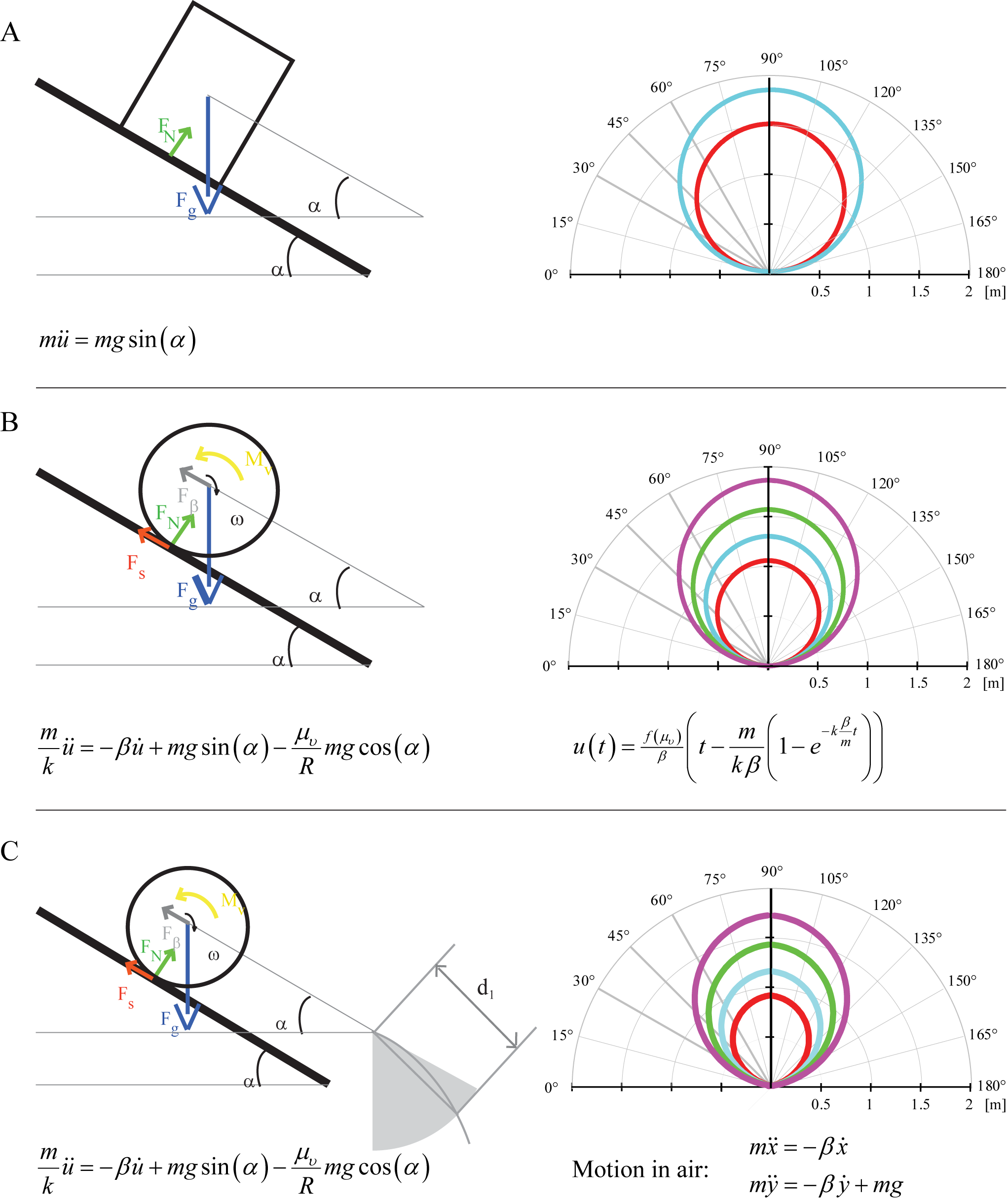

Supplement: Figure S2 — Schematic illustration of different types of falls from an inclined plane. A. Sliding of a parallelepiped under gravity on a friction-less plane. B. Rolling without slipping of a sphere under gravity and air drag linear in speed. C. As in B, followed by free-fall under gravity and air drag. Left panels: free-body diagrams of falls. α: inclination angle relative to the horizontal. FN: ground reaction force. Fg: gravitational force. Fβ: air resistance force. Fs: sliding resistance. Mv: rolling resistance moment. In C, d1 corresponds to the arrival point after free-fall (as in Fig. S1). Right panels: isochronous lines for falls at different tilt angles. Each isoline connects the x,y starting positions yielding the same duration of fall along planes of different inclinations: red for 550 ms, cyan for 610 ms, green for 670 ms, and purple for 730 ms. In A, the isolines corresponding to 670 and 730 ms are not drawn because out-of-scale for higher tilt angles. In C, the isolines have been computed for d1 = 0.15 m. (TIF) [file pone.0099837.s002.tif]
