# Supplementary material for: Neural Extrapolation of Motion for a Ball Rolling Down an Inclined Plane
Source: PLoS One. 2014 Jun 18;9(6):e99837. doi: 10.1371/journal.pone.0099837 (PMC4062474; doi:10.1371/journal.pone.0099837)
Supplement: Table S1 — Ball motion parameters in Experiment 1 (Incline and Air) and Experiment 2 (Incline). (DOCX) [file pone.0099837.s003.docx]

|  |  | **Incline** | | | **Air** | | |
| --- | --- | --- | --- | --- | --- | --- | --- |
| **Angle** | **nBMD** | **distance**  **(u axis)** | **speed at lower end**  **(u axis)** | **time** | **distance**  **d1 (x_1_ ; y_1_)** | **speed at nIP**  **v (v_x_ ; v_y_)** | **time** |
| **[°]** | **[ms]** | **[m]** | **[ m·s^-1^]** | **[ms]** | **[m]** | **[ m·s^-1^]** | **[ms]** |
| 30 | 550 | 0.36 | 1.51 | 470.60 | 0.13 (0.10 ; 0.09) | 2.02 (1.31 ; 1.54) | 80.24 |
| 30 | 610 | 0.46 | 1.71 | 533.98 | 0.14 (0.11 ; 0.09) | 2.18 (1.48 ; 1.60) | 76.20 |
| 30 | 670 | 0.58 | 1.92 | 599.20 | 0.14 (0.11 ; 0.09) | 2.35 (1.66 ; 1.66) | 70.94 |
| 30 | 730 | 0.70 | 2.12 | 662.36 | 0.15 (0.12 ; 0.09) | 2.52 (1.84 ; 1.73) | 68.13 |
| 45 | 550 | 0.61 | 2.40 | 509.52 | 0.11 (0.07 ; 0.08) | 2.70 (1.70 ; 2.10) | 41.26 |
| 45 | 610 | 0.77 | 2.70 | 572.74 | 0.11 (0.07 ; 0.08) | 2.97 (1.91 ; 2.28) | 37.86 |
| 45 | 670 | 0.95 | 3.00 | 636.00 | 0.11 (0.07 ; 0.08) | 3.24 (2.12 ; 2.45) | 34.26 |
| 45 | 730 | 1.15 | 3.29 | 698.32 | 0.11 (0.07 ; 0.08) | 3.52 (2.33 ; 2.64) | 31.78 |
| 60 | 550 | 0.82 | 3.1 | 528.86 | 0.08 (0.04 ; 0.07) | 3.32 (1.56 ; 2.93) | 23.70 |
| 60 | 610 | 1.02 | 3.47 | 589.12 | 0.08 (0.04 ; 0.07) | 3.66 (1.74 ; 3.22) | 21.52 |
| 60 | 670 | 1.25 | 3.83 | 650.46 | 0.08 (0.04 ; 0.07) | 4.00 (1.92 ; 3.52) | 19.78 |
| 60 | 730 | 1.49 | 4.2 | 711.86 | 0.08 (0.04 ; 0.07) | 4.36 (2.10 ; 3.81) | 18.14 |

Table S1
