# Supplementary material for: Neural Extrapolation of Motion for a Ball Rolling Down an Inclined Plane
Source: PLoS One. 2014 Jun 18;9(6):e99837. doi: 10.1371/journal.pone.0099837 (PMC4062474; doi:10.1371/journal.pone.0099837)
Supplement: Table S2 — Square-root of the eigenvalues of 95% tolerance ellipses in Experiment 1. They correspond to the semi-axes of the ellipses (cm). *Eigenvalues not statistically distinct. (DOCX) [file pone.0099837.s004.docx]

|  |  | **nBMD [ms]** | | | |
| --- | --- | --- | --- | --- | --- |
| **Angle [°]** |  | **550** | **610** | **670** | **730** |
| 30 | Minor | 4.71 | 5.09 | 4.99 | 5.02* |
|  | Major | 6.76 | 7.14 | 7.11 | 6.87* |
| 45 | Minor | 5.28 | 5.30 | 4.66 | 5.37 |
|  | Major | 8.49 | 7.83 | 8.53 | 8.67 |
| 60 | Minor | 5.17 | 4.90 | 5.64 | 5.63 |
|  | Major | 8.90 | 9.41 | 9.22 | 11.22 |

**Table S2**
