# Supplementary material for: Neural Extrapolation of Motion for a Ball Rolling Down an Inclined Plane
Source: PLoS One. 2014 Jun 18;9(6):e99837. doi: 10.1371/journal.pone.0099837 (PMC4062474; doi:10.1371/journal.pone.0099837)
Supplement: Table S3 — Orientation (in degrees) of the major axis of 95% tolerance ellipses in Experiment 1. (DOCX) [file pone.0099837.s005.docx]

|  | **nBMD [ms]** | | | |
| --- | --- | --- | --- | --- |
| **Angle [°]** | **550** | **610** | **670** | **730** |
| 30 | 42.03 | 44.38 | 34.38 | 53.50 |
| 45 | 55.46 | 52.70 | 51.11 | 60.13 |
| 60 | 69.64 | 78.22 | 79.04 | 81.18 |

**Table S3.**
