# Supplementary material for: Neural Extrapolation of Motion for a Ball Rolling Down an Inclined Plane
Source: PLoS One. 2014 Jun 18;9(6):e99837. doi: 10.1371/journal.pone.0099837 (PMC4062474; doi:10.1371/journal.pone.0099837)
Supplement: Table S4 — Mean values and standard deviations (SD) of the kinematical variables MD, ISpeed, PSpeed, Speed_IP and TCurv as a function of the four nBMDs and the three incline tilting angles in Experiment 1. (DOCX) [file pone.0099837.s006.docx]

|  |  |  | **nBMD [ms]** | | | |
| --- | --- | --- | --- | --- | --- | --- |
|  | **Angle [°]** |  | **550** | **610** | **670** | **730** |
| **TCurv**  **[mm]** | 30 | Mean | 15.79 | 15.73 | 15.98 | 17.79 |
|  |  | SD | 8.19 | 7.71 | 7.94 | 8.51 |
|  | 45 | Mean | 16.65 | 16.88 | 17.11 | 17.91 |
|  |  | SD | 8.76 | 7.57 | 8.59 | 9.46 |
|  | 60 | Mean | 17.68 | 17.97 | 17.98 | 18.46 |
|  |  | SD | 10.50 | 10.49 | 9.27 | 10.09 |
| **MD [ms]** | 30 | Mean | 280.08 | 282.51 | 293.43 | 293.22 |
|  |  | SD | 67.41 | 63.27 | 80.05 | 74.95 |
|  | 45 | Mean | 274.57 | 283.76 | 285.19 | 296.65 |
|  |  | SD | 54.93 | 57.81 | 65.33 | 73.53 |
|  | 60 | Mean | 271.31 | 274.65 | 284.31 | 280.48 |
|  |  | SD | 60.16 | 69.19 | 70.29 | 77.82 |
| **PSpeed**  **[m·s^-1^]** | 30 | Mean | 2.61 | 2.60 | 2.61 | 2.67 |
|  |  | SD | 0.58 | 0.57 | 0.56 | 0.60 |
|  | 45 | Mean | 2.62 | 2.61 | 2.65 | 2.68 |
|  |  | SD | 0.55 | 0.56 | 0.54 | 0.54 |
|  | 60 | Mean | 2.69 | 2.66 | 2.73 | 2.77 |
|  |  | SD | 0.66 | 0.67 | 0.66 | 0.63 |
| **Speed_IP [m·s^-1^]** | 30 | Mean | 2.42 | 2.39 | 2.41 | 2.46 |
|  |  | SD | 0.62 | 0.63 | 0.61 | 0.66 |
|  | 45 | Mean | 2.31 | 2.38 | 2.43 | 2.43 |
|  |  | SD | 0.72 | 0.69 | 0.67 | 0.71 |
|  | 60 | Mean | 2.39 | 2.38 | 2.43 | 2.49 |
|  |  | SD | 0.80 | 0.79 | 0.81 | 0.76 |
| **ISpeed**  **[m·s^-1^]** | 30 | Mean | 0.21 | 0.21 | 0.19 | 0.20 |
|  |  | SD | 0.14 | 0.15 | 0.14 | 0.14 |
|  | 45 | Mean | 0.21 | 0.18 | 0.20 | 0.17 |
|  |  | SD | 0.16 | 0.11 | 0.18 | 0.13 |
|  | 60 | Mean | 0.23 | 0.22 | 0.20 | 0.22 |
|  |  | SD | 0.17 | 0.14 | 0.14 | 0.15 |

**Table S4.**
