# Supplementary material for: Neural Extrapolation of Motion for a Ball Rolling Down an Inclined Plane
Source: PLoS One. 2014 Jun 18;9(6):e99837. doi: 10.1371/journal.pone.0099837 (PMC4062474; doi:10.1371/journal.pone.0099837)
Supplement: Table S5 — Square-root of the eigenvalues of 95% tolerance ellipses in Experiment 2. They correspond to the semi-axes of the ellipses (cm). *Eigenvalues not statistically distinct. (DOCX) [file pone.0099837.s007.docx]

|  |  | **nBMD [ms]** | | | |
| --- | --- | --- | --- | --- | --- |
| **Angle [°]** |  | **550** | **610** | **670** | **730** |
| 30 | Minor | 5.01 | 6.01 | 5.37 | 5.99 |
|  | Major | 8.35 | 8.45 | 8.32 | 8.39 |
| 45 | Minor | 6.97* | 6.68* | 5.96 | 6.62 |
|  | Major | 9.14* | 8.91* | 9.63 | 9.78 |
| 60 | Minor | 6.99* | 6.86* | 7.26* | 8.17* |
|  | Major | 9.08* | 8.84* | 8.56* | 9.25* |

**Table S5.**
