# Supplementary material for: Neural Extrapolation of Motion for a Ball Rolling Down an Inclined Plane
Source: PLoS One. 2014 Jun 18;9(6):e99837. doi: 10.1371/journal.pone.0099837 (PMC4062474; doi:10.1371/journal.pone.0099837)
Supplement: Table S6 — Inclination (in degree) of the major axis of 95% tolerance ellipses in Experiment 2. (DOCX) [file pone.0099837.s008.docx]

|  | **nBMD [ms]** | | | |
| --- | --- | --- | --- | --- |
| **Angle**  **[°]** | **550** | **610** | **670** | **730** |
| 30 | 104.01 | 100.78 | 105.77 | 110.09 |
| 45 | 148.77* | 137.26 | 149.36 | 153.46 |
| 60 | 130.99* | 141.20* | 145.27* | 134.94* |

**Table S6.**
