# Supplementary material for: Neural Extrapolation of Motion for a Ball Rolling Down an Inclined Plane
Source: PLoS One. 2014 Jun 18;9(6):e99837. doi: 10.1371/journal.pone.0099837 (PMC4062474; doi:10.1371/journal.pone.0099837)
Supplement: Table S7 — Mean values and standard deviations (SD) of the kinematical variables MD, ISpeed, PSpeed, Speed_IP and TCurv as a function of the four nBMDs and the three incline tilting angles in Experiment 2. (DOCX) [file pone.0099837.s009.docx]

|  |  |  | **nBMD [ms]** | | | |
| --- | --- | --- | --- | --- | --- | --- |
|  | **Angle [°]** |  | **550** | **610** | **670** | **730** |
| **MD [ms]** | 30 | Mean | 222.83 | 223.75 | 225.32 | 225.69 |
|  |  | SD | 53.93 | 57.14 | 51.52 | 52.77 |
|  | 45 | Mean | 224.62 | 221.02 | 223.06 | 228.57 |
|  |  | SD | 63.25 | 57.60 | 62.53 | 67.23 |
|  | 60 | Mean | 231.44 | 233.08 | 234.08 | 236.12 |
|  |  | SD | 60.38 | 63.53 | 66.86 | 73.34 |
| **ISpeed [m·s^-1^]** | 30 | Mean | 0.48 | 0.49 | 0.44 | 0.42 |
|  |  | SD | 0.26 | 0.27 | 0.24 | 0.26 |
|  | 45 | Mean | 0.50 | 0.48 | 0.48 | 0.46 |
|  |  | SD | 0.31 | 0.29 | 0.30 | 0.32 |
|  | 60 | Mean | 0.41 | 0.41 | 0.43 | 0.40 |
|  |  | SD | 0.24 | 0.24 | 0.28 | 0.27 |
| **PSpeed [m·s^-1^]** | 30 | Mean | 2.12 | 2.10 | 2.15 | 2.17 |
|  |  | SD | 0.57 | 0.58 | 0.57 | 0.57 |
|  | 45 | Mean | 2.14 | 2.15 | 2.15 | 2.23 |
|  |  | SD | 0.56 | 0.56 | 0.55 | 0.59 |
|  | 60 | Mean | 2.11 | 2.14 | 2.18 | 2.22 |
|  |  | SD | 0.56 | 0.59 | 0.57 | 0.56 |
| **Speed_IP [m·s^-1^]** | 30 | Mean | 1.76 | 1.77 | 1.87 | 1.92 |
|  |  | SD | 0.73 | 0.72 | 0.69 | 0.70 |
|  | 45 | Mean | 1.75 | 1.81 | 1.84 | 1.94 |
|  |  | SD | 0.74 | 0.70 | 0.70 | 0.77 |
|  | 60 | Mean | 1.79 | 1.83 | 1.85 | 1.91 |
|  |  | SD | 0.71 | 0.72 | 0.74 | 0.74 |
| **TCurv [mm]** | 30 | Mean | 8.87 | 9.35 | 8.99 | 8.87 |
|  |  | SD | 5.08 | 4.60 | 4.79 | 4.09 |
|  | 45 | Mean | 9.68 | 9.70 | 9.54 | 9.47 |
|  |  | SD | 4.80 | 4.72 | 4.43 | 4.32 |
|  | 60 | Mean | 8.89 | 8.19 | 8.28 | 8.99 |
|  |  | SD | 5.04 | 4.64 | 4.62 | 5.19 |

**Table S7.**
