# Supplementary material for: Neural Extrapolation of Motion for a Ball Rolling Down an Inclined Plane
Source: PLoS One. 2014 Jun 18;9(6):e99837. doi: 10.1371/journal.pone.0099837 (PMC4062474; doi:10.1371/journal.pone.0099837)
Supplement: Table S8 — Ball motion parameters in Experiment 3. (DOCX) [file pone.0099837.s010.docx]

|  | **Incline** | | |
| --- | --- | --- | --- |
| **Angle** | **distance**  **(u axis)** | **speed at lower end**  **(u axis)** | **time** |
| **[°]** | **[m]** | **[m·s^-1^]** | **[ms]** |
| 30 | 0.36 | 1.51 | 471 |
| 30 | 0.58 | 1.92 | 599 |
| 30 | 1.15 | 2.67 | 862 |
| 30 | 1.49 | 3.04 | 982 |
| 45 | 0.61 | 2.40 | 510 |
| 45 | 0.77 | 2.7 | 573 |
| 45 | 0.95 | 3 | 636 |
| 45 | 1.49 | 3.75 | 796 |
| 60 | 0.612 | 2.69 | 456 |
| 60 | 0.82 | 3.12 | 529 |
| 60 | 1.25 | 3.83 | 650 |
| 60 | 1.49 | 4.2 | 712 |

Table S8.
