# Supplementary material for: Neural Extrapolation of Motion for a Ball Rolling Down an Inclined Plane
Source: PLoS One. 2014 Jun 18;9(6):e99837. doi: 10.1371/journal.pone.0099837 (PMC4062474; doi:10.1371/journal.pone.0099837)
Supplement: Table S9 — Experiment 3. Mean values and standard deviations (SD) of the kinematical variables: time interval between OT and ball stop time (BST), PSpeed, Interval time between PSpeed time and BST as a function of the four ball motion duration for each incline tilt and the three incline tilting angles. Note that ball motion duration time1, time2, time3, time4 were not the same for the three incline tilting angles. (DOCX) [file pone.0099837.s011.docx]

|  |  | **Motion duration on the incline** | | | | |
| --- | --- | --- | --- | --- | --- | --- |
|  | **Angle [°]** |  | **time1** | **time2** | **time3** | **time4** |
| **OT - BST**  **[ms]** | 30 | Mean | -131.27 | -160.36 | -183.86 | -178.24 |
|  |  | SD | 70.82 | 85.27 | 119.68 | 93.91 |
|  | 45 | Mean | -163.09 | -171.84 | -174.97 | -190.43 |
|  |  | SD | 79.18 | 97.65 | 100.38 | 134.96 |
|  | 60 | Mean | -130.56 | -144.33 | -157.14 | -133.52 |
|  |  | SD | 70.12 | 63.45 | 101.92 | 91.63 |
| **PSpeed [m·s^-1^]** | 30 | Mean | 1.73 | 1.84 | 1.96 | 2.12 |
|  |  | SD | 0.72 | 0.74 | 0.80 | 0.76 |
|  | 45 | Mean | 1.75 | 1.81 | 1.90 | 2.10 |
|  |  | SD | 0.56 | 0.60 | 0.62 | 0.71 |
|  | 60 | Mean | 2.12 | 2.22 | 2.35 | 2.45 |
|  |  | SD | 0.72 | 0.77 | 0.82 | 0.84 |
| **Time interval between PSpeed time and BST**  **[ms]** | 30 | Mean | 89.53 | 65.19 | 40.12 | 28.08 |
|  |  | SD | 80.79 | 85.93 | 90.27 | 88.61 |
|  | 45 | Mean | 76.55 | 70.05 | 78.66 | 51.14 |
|  |  | SD | 103.09 | 114.19 | 103.03 | 85.70 |
|  | 60 | Mean | 68.99 | 49.56 | 39.24 | 60.01 |
|  |  | SD | 80.88 | 78.21 | 103.31 | 121.06 |

**Table S9.**
